# Epicoccin A Ameliorates PD-like Symptoms in Zebrafish: Enhancement of PINK1/Parkin-Dependent Mitophagy and Inhibition of Excessive Oxidative Stress

**DOI:** 10.3390/md23040175

**Published:** 2025-04-17

**Authors:** Haicheng Ye, Dan Li, Lei Zhang, Yufei Wang, Cong Wang, Meng Jin, Houwen Lin, Peihai Li, Chen Sun, Ning Li

**Affiliations:** 1 Shandong Academy of Sciences & Engineering Research Center of Zebrafish Models for Human Diseases and Drug Screening of Shandong Province, Biology Institute, Qilu University of Technology, Jinan 250000, China; yhc108118@163.com (H.Y.); lid00414@163.com (D.L.); mjin1985@hotmail.com (M.J.); liph@sdas.org (P.L.); mornings0123@163.com (C.S.); 2 Shandong Overseas Fisheries Association, Jinan 250000, China; sddwfadmin@163.com; 3 Key Laboratory of Chemistry and Engineering of Forest Products, State Ethnic Affairs Commission & Guangxi Key Laboratory of Chemistry and Engineering of Forest Products/Guangxi Collaborative Innovation Center for Chemistry and Engineering of Forest Products, Guangxi Minzu University, Nanning 530000, China; wyf19991126@126.com (Y.W.); wangcong123206@163.com (C.W.); 4 Research Center for Marine Drugs, State Key Laboratory of Oncogenes and Related Genes, Department of Pharmacy, School of Medicine, Shanghai Jiao Tong University, Shanghai 200000, China; franklin67@126.com

**Keywords:** neuroprotective effect, marine-derived fungus, MPTP, α-synuclein, ROS, transcriptome analysis, molecular docking, underlying mechanism

## Abstract

Parkinson’s disease (PD) is the second most prevalent neurodegenerative disorder, yet effective agents for its prevention and therapy remain highly limited. Epicoccin A, a significant secondary metabolite from *Exserohilum* sp., demonstrates various biological activities; however, its neuroprotective effects have not been elucidated. Here, we investigated the therapeutic potential of epicoccin A for PD by evaluating its impact on neural phenotype, reactive oxygen species (ROS) generation, and locomotor activity in PD-like zebrafish. Transcriptomic analysis and molecular docking were conducted, with key gene expressions further verified using real-time qPCR. As a result, epicoccin A notably mitigated dopaminergic neuron loss, neural vasculature deficiency, nervous system injury, ROS accumulation, locomotor impairments, and abnormal expressions of hallmark genes associated with PD and oxidative stress. Underlying mechanism investigation indicated epicoccin A may alleviate PD-like symptoms by activating PINK1/Parkin-dependent mitophagy, as evidenced by the reversal of aberrant gene expressions related to the pink1/parkin pathway and its upstream mTOR/FoxO pathway following epicoccin A co-treatments. This finding was further confirmed by the robust interactions between epicoccin A and these mitophagy regulators. Our results suggest that epicoccin A relieves PD symptoms by activating pink1/parkin-dependent mitophagy and inhibiting excessive oxidative stress, highlighting its potential as a therapeutic approach for PD.

## 1. Introduction

Parkinson’s disease (PD) is the second most common neurodegenerative disease, yet it currently lacks effective treatment options [1]. The pathology of PD is typically characterized by a progressive loss of dopaminergic (DA) neurons in the substantia nigra pars compacta, accompanied by the accumulation of Lewy bodies (LBs) and Lewy neurites in neurons, in which fibrillar α-synuclein aggregates constitute the primary protein component [2,3]. The clinical manifestations of PD encompass both motor symptoms and non-motor symptoms [4]. The nigrostriatal pathway is implicated in voluntary movement coordination [5]. A 70–80% loss of dopamine in the striatum will significantly impair the nigrostriatal pathway, leading to typical motor symptoms such as bradykinesia, rigidity, resting tremors, gait dysfunction, and postural instability [6,7]. Non-motor symptoms, including reduced concentration, REM-sleep behavior disorder, and hyposmia, often precede motor symptoms by several years, complicating early diagnosis and intervention [8]. As a chronic condition that potentially spans decades, PD imposes substantial lifetime burdens on both patients and healthcare costs [9].

Until now, PD has remained incurable, largely attributed to its complex pathogenesis and unclear etiology. Currently, existing treatments including dopamine replacement therapies (e.g., levodopa), dopamine receptor agonists, monoamine oxidase B inhibitors, and neuroprotectants, can alleviate the symptoms of patients but fail to halt disease progression or reverse disabilities [10,11]. Moreover, these therapeutic agents exhibit severe side effects following long-term administration, or unfavorable biochemical and pharmacokinetic properties, highlighting the necessity of developing new drugs with minimal side effects for PD therapy [12]. Accumulating evidence indicates that α-synuclein misfolding and aggregation, oxidative stress, mitochondrial dysfunction, neuroinflammation, and neuronal apoptosis are implicated in the progression of neuronal damage in PD [13]. It is noteworthy that the disruption of the mitophagy process impairs mitochondrial potential and respiratory chain function, leading to the accumulation of reactive oxygen species (ROS) and ultimately resulting in DA neuron damage [14]. The aggregation of α-synuclein is also considered as a consequence of impaired mitophagy [15,16]. Thus, targeting the improvement of impaired mitophagy and modulating aberrant oxidative stress may offer a potential therapeutic strategy.

**Figure 1:**
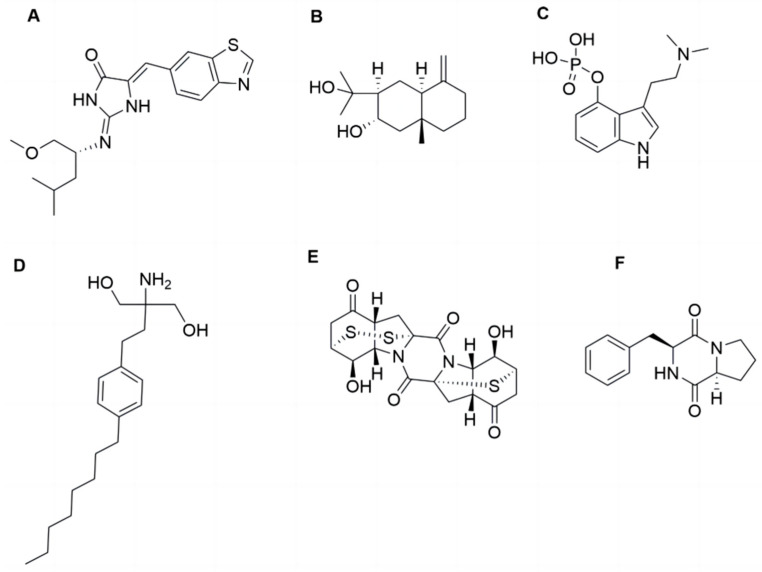
The structures of epicoccin A and the compounds mentioned. (**A**) Leucettinib-21. (**B**) Arctiol. (**C**) Psilocybin. (**D**) Fingolimod. (**E**) Epicoccin A. (**F**) Cyclo-(L-Pro-L-Phe).

Natural products, particularly those derived from marine environments, have been extensively investigated due to their potent biological activities and minimal side effects [17]. Neuroprotective compounds have been identified from marine natural products. For example, Leucettinib-21, derived from *Leucetta microraphis*, ameliorates Alzheimer’s disease by inhibiting DYRK1A kinase and is currently in phase I clinical trials. In addition, compounds derived from fungi, characterized by their complex structures, have also emerged as an important source for the discovery of neuroprotective active substances [18,19]. For instance, arctiol, isolated from the fungus *Eutypella* sp. F0219, could effectively inhibit neuroinflammation by suppressing the NF-κB pathway and macrophage polarization [20]. Psilocybin, a fungal secondary metabolite primarily derived from Basidiomycota, can exert neuroregulatory activity through modulating neurotransmitter release and synaptic plasticity. Psilocybin is currently in phase II clinical trials for treating PD-related depression [21]. Fingolimod, a structurally modified compound of myriocin derived from the fugus *Cordyceps sinensis*, can mitigate neuronal oxidative stress by activating Akt/ERK signaling pathway. To date, however, no neuroprotective activities have been reported for the secondary metabolites derived from the fungi *Exserohilum* sp., which is distributed in environments including deep-sea sediments [22]. Among its secondary metabolites, epicoccin A is a sulfur bridge-containing diketopiperazine compound [23] (Figure 1). Previous studies have indicated that Cyclo-(L-Pro-L-Phe), a diketopiperazine compound isolated from the marine fungus *Aspergillus versicolor*, demonstrated potential neuroprotective activity against oxidative stress-induced neurodegeneration in SH-SY5Y cells [24]. However, the documented activities of epicoccin A are currently limited to anti-inflammatory, antimicrobial, and anticancer effects, with no investigations into its neuroprotective potential available. Inflammation inhibition can also improve mitophagy dysfunction, facilitating the protection of DA neurons and thereby potentially mitigating disease progression [25,26]. Furthermore, the structure of epicoccin A is more complex than that of Cy-clo-(L-Pro-L-Phe) and contains both sulfur and nitrogen elements, which generally endow compounds with drug-like potential [27]. Therefore, epicoccin A may hold the protective potential against PD and warrants further exploration.

The zebrafish (*Danio rerio*), a freshwater vertebrate, has become a valuable model for investigating central nervous system (CNS) disorders such as Alzheimer’s disease [28] and PD [29], as well as for the development of associated therapeutic agents. The neural circuits and functions in zebrafish brains, which include ventral diencephalon and DA neurons, closely resemble those of mammals. Zebrafish harbor functionally conserved genes orthologous to those implicated in PD. Furthermore, the development of the DA system in zebrafish is almost completed by 96 hours post-fertilization (hpf), and the optical transparency of larvae allows for the precise observation of neuronal changes [30]. The neurotoxin 1-methyl-4-phenyl-1,2,3,6-tetrahydropyridine (MPTP) can selectively induce degeneration of DA neurons in the substantia nigra by inhibiting mitochondrial complex I activity through its metabolite MPP^+^ [31]. Exposure to MPTP results in phenotypes in zebrafish that are similar to those observed in mammals and humans, such as the selective loss of DA neurons and altered locomotor behavior [32]. Thus, the MPTP-induced PD model in zebrafish has been widely applied for screening anti-PD compounds and investigating underlying molecular mechanisms [33,34]. In this study, we assessed the neuroprotective effect of epicoccin A using the MPTP-induced PD-like zebrafish model and explored the underlying mechanisms contributing to its anti-PD activity.

## 2. Results

### 2.1. Effect of Epicoccin A on the Loss of DA Neurons in PD

**Figure 2:**
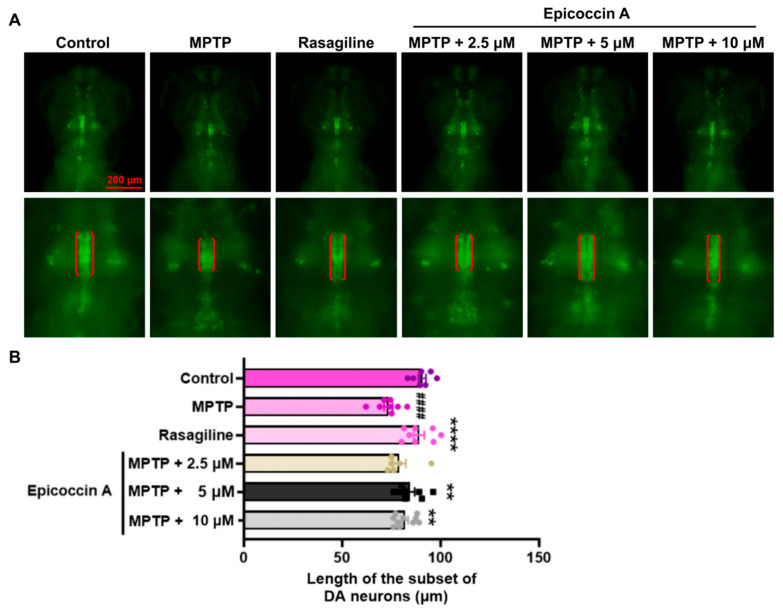
Remissive effect of epicoccin A on the loss of DA neurons in PD. (**A**) Representative fluorescent images of *slc18a2:GFP* zebrafish, where the length of the subset of DA neurons analyzed were denoted by the red brackets. Scale bar, 200 μm. (**B**) Statistical analysis of the length of the subset of DA neurons in each group, n = 8. The data are presented as mean ± SEM; ^####^ *p* < 0.0001 compared to the control group; ** *p* < 0. 01 and **** *p* < 0.0001 compared to the MPTP group.

An initial toxicity assessment was conducted by treating zebrafish exclusively with six different concentrations (2.5, 5, 10, 15, 20, and 30 μM) of epicoccin A individually (Supplementary Figure S1). We observed that treatment with 30 μM epicoccin A alone led to pericardial and yolk sac edema of zebrafish at 72 hpf and 96 hpf, followed by complete mortality at 120 hpf. Therefore, we selected concentrations below 30 μM, namely 2.5, 5, and 10 μM, to further investigate the neuroprotective activity of epicoccin A. To investigate the inhibitory effect of epicoccin A on the loss of DA neurons in PD, we evaluated the length change in DA neurons using transgenic zebrafish *slc18a2:GFP*. As a result, zebrafish at 96 hpf after being treated with MPTP exhibited a reduced length in the subset of DA neurons (Figure 2), consistent with previous findings [28]. In contrast, the epicoccin A plus MPTP co-treatments significantly reversed the reduction, a finding consistent with the result observed in the rasagiline co-treatment. This suggests epicoccin A may play a neuroprotective role against PD.

### 2.2. Effect of Epicoccin A on Nervous System Injury in PD

**Figure 3:**
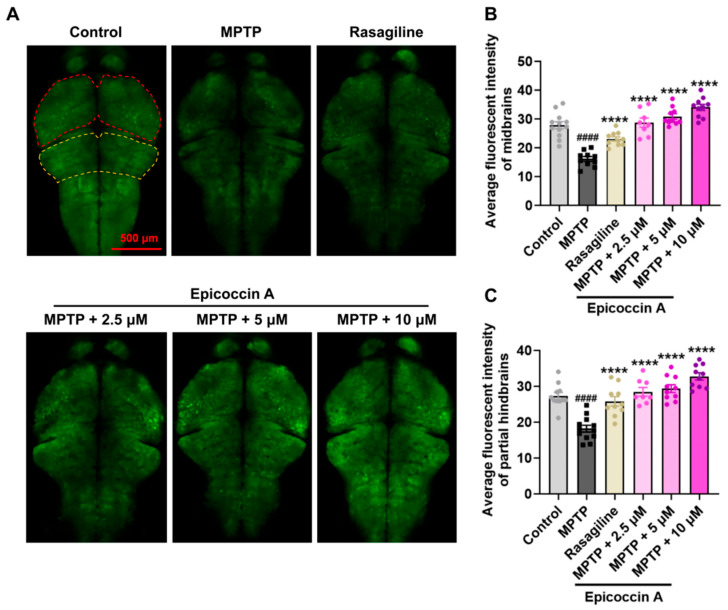
The inhibitory effect of epicoccin A on MPTP-induced nervous system injury in zebrafish brains. (**A**) Representative fluorescent microscopy images of *elavl3:EGFP* zebrafish from the control, MPTP, rasagiline, and epicoccin A plus MPTP co-treatment groups. Scale bar, 500 μm. (**B**) Statistical analysis of the average fluorescent intensity in the midbrain regions (as indicated by red dotted lines) of zebrafish in each group, n = 8. (**C**) Statistical analysis of the average fluorescent intensity in the partial hindbrain regions (as indicated by yellow dotted lines) of zebrafish in each group, n = 8. The data are presented as mean ± SEM; ^####^ *p* < 0.0001 compared to the control group; **** *p* < 0.0001 compared to the MPTP group.

Given that PD is characterized by α-synucleinopathy affecting the CNS, we examined the impact of epicoccin A on nervous system damage in the brains of PD-affected *Tg* (*elavl3:EGFP*) zebrafish, a line with fluorescent-labeled neurons. We found the average fluorescent intensity in the midbrain, partial hindbrain, and even the entire brain was notably reduced in zebrafish treated with MPTP as compared to the control (Figure 3). In contrast, co-treatment with epicoccin A significantly prevented MPTP-induced loss of fluorescent intensity in the midbrain and partial hindbrain regions, suggesting the restored effect of epicoccin A on the injuries of neurons and the nervous system in PD.

### 2.3. Effect of Epicoccin A on Neural Vasculature Loss in PD

**Figure 4:**
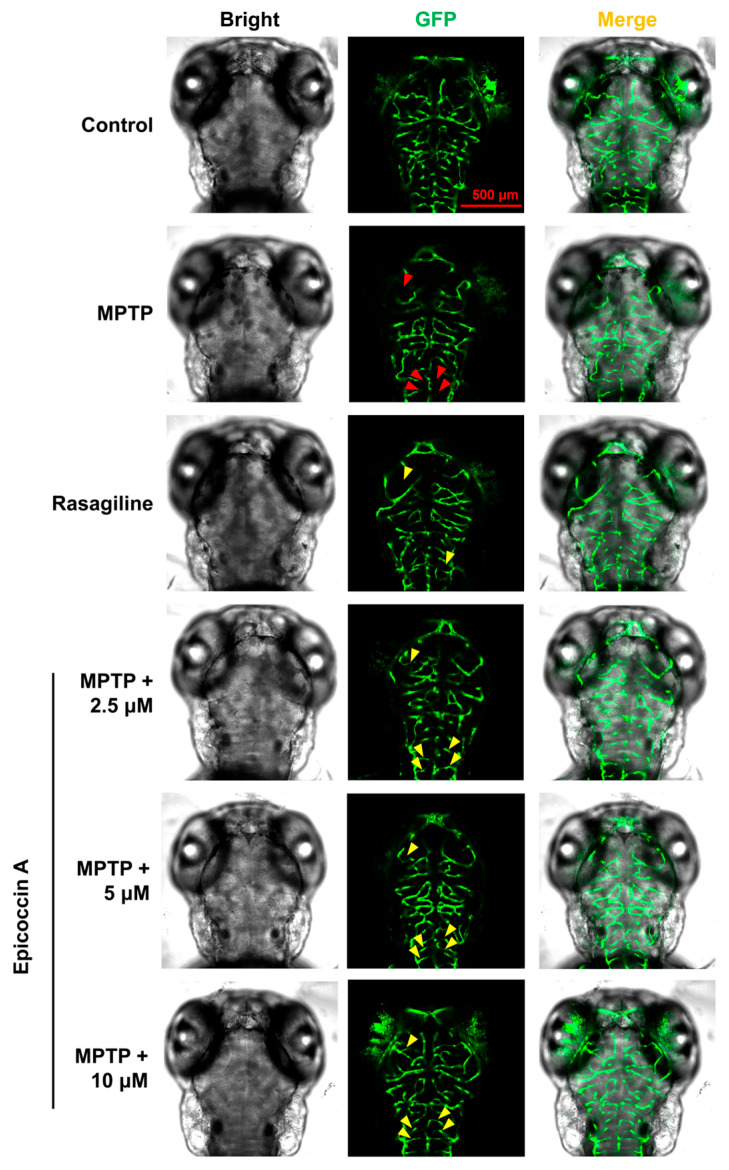
Ameliorative effect of epicoccin A on MPTP-induced loss and disorganization of neural vasculature. Representative fluorescent microscopy images of *flk1:GFP* zebrafish from the control, MPTP, rasagiline, and epicoccin A plus MPTP co-treatments. Red arrows indicated the loss of neural vasculature induced by MPTP. Yellow arrows indicated the unmarred or incompletely injured neural vasculature as compared with the MPTP treatment. Scale bar, 500 μm.

To further evaluate the role of epicoccin A in mitigating PD, we investigated the alteration of neuronal vasculature loss induced by MPTP after co-treatment with epicoccin A. The results indicated that exposure to MPTP resulted in a pronounced loss and disorganization of vascular structure in zebrafish brains (indicated by red arrows in Figure 4), consistent with previous findings [35]. Contrarily, epicoccin A plus MPTP co-treatment significantly alleviated the loss and disorganization of the neural vasculature (indicated by yellow arrows), indicating that epicoccin A plays a significant role in maintaining the integrity of neural vasculature.

### 2.4. Effect of Epicoccin A on Generation of ROS in Zebrafish PD Model

**Figure 5:**
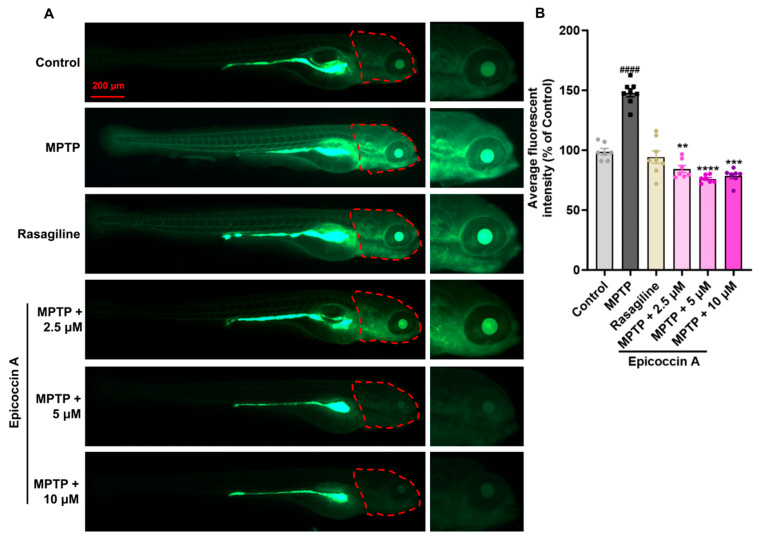
The inhibitory effect of epicoccin A on ROS overproduction in the brains of zebrafish with PD. (**A**) Fluorescent images depicting ROS levels in zebrafish larvae from control, MPTP, rasagiline, and epicoccin A plus MPTP co-treatments. Enlarged images are provided for clear visualization. Scale bar, 200 µm. (**B**) Quantification of fluorescent intensity representing ROS levels in the brains (as indicated by red dotted lines) of zebrafish larvae in each group, n = 8. The data are presented as mean ± SEM; ^####^ *p* < 0.0001 compared to the control group; ** *p* < 0.01, *** *p* < 0.001, and **** *p* < 0.0001 compared to the MPTP group.

The accumulation of ROS is closely linked to the pathogenesis of PD, as it contributes to oxidative stress and neuronal damage [35]. Following MPTP treatment, the fluorescent intensity in zebrafish brains at 120 hpf significantly increased as compared to the control group, suggesting an overproduction of ROS in PD-like zebrafish (Figure 5). Contrarily, epicoccin A co-treatments effectively inhibited the ROS overproduction, as evidenced by the notable decrease in fluorescent intensity in zebrafish brains. This suggests that epicoccin A could mitigate the oxidative stress in the brains of PD-like zebrafish.

### 2.5. Effect of Epicoccin A on Locomotor Impairment in PD

**Figure 6:**
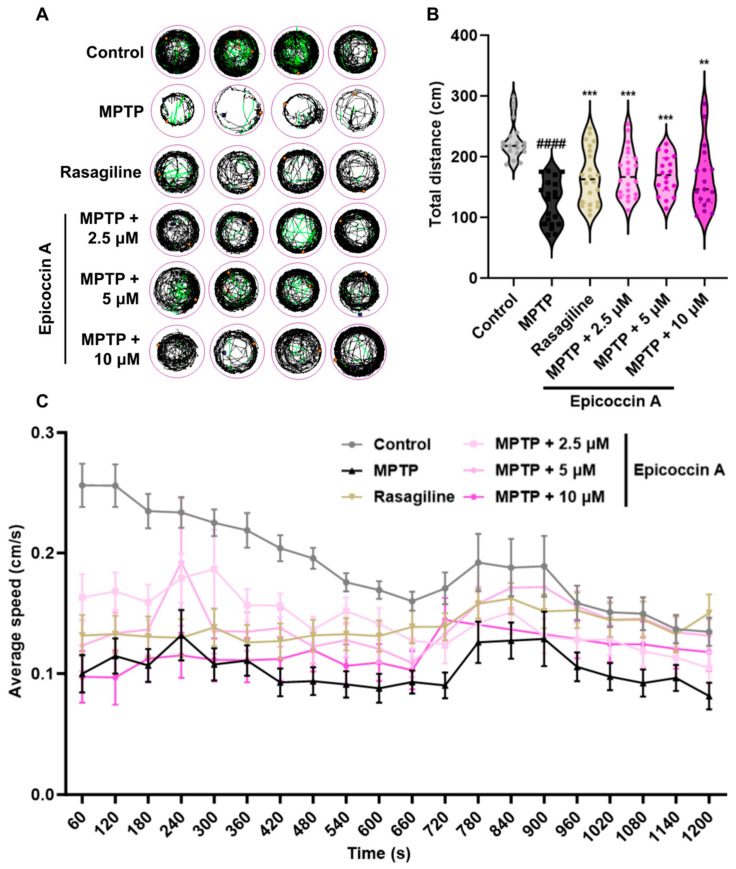
Improved effect of epicoccin A on MPTP-induced locomotor impairment in zebrafish. (**A**) Four representative movement trajectories of zebrafish from control, MPTP, rasagiline, and epicoccin A plus MPTP co-treatments, n = 22. Red, green, and black lines represent fast (>0.5 cm/s), medium (0.2–0.5 cm/s), and slow (<0.2 cm/s) movement trajectories, respectively. (**B**) The total distance moved by zebrafish, n = 22. (**C**) Average speed was calculated at every 60 s interval within the 20 min recording period for all individuals from each group, n = 22. The data are presented as the mean ± SEM; ^####^ *p* < 0.0001 compared to the control group; ** *p* < 0.01 and *** *p* < 0.001 compared to the MPTP group.

To evaluate whether epicoccin A can alleviate locomotor deficits associated with PD, we performed behavioral assessment at 120 hpf, the earliest time point at which zebrafish exhibit maximal spontaneous locomotion [35]. The behavioral tracks of zebrafish were recorded, and quantitative analysis was conducted as shown in Figure 6. Consistent with previous studies [36,37], MPTP treatment significantly reduced zebrafish mobility, as evidenced by a significant decrease in movement trajectories, total swimming distance, and average speed. In contrast, co-treatments with epicoccin A significantly increased the total distance traveled, along with a notable elevation in the average speed and movement trajectories, comparable to those observed in the rasagiline group. These findings suggest that epicoccin A may alleviate the PD-like locomotor deficits in zebrafish.

### 2.6. Effects of Epicoccin A on the Abnormal Expressions of Genes Related to Neurodevelopment and PD

**Figure 7:**
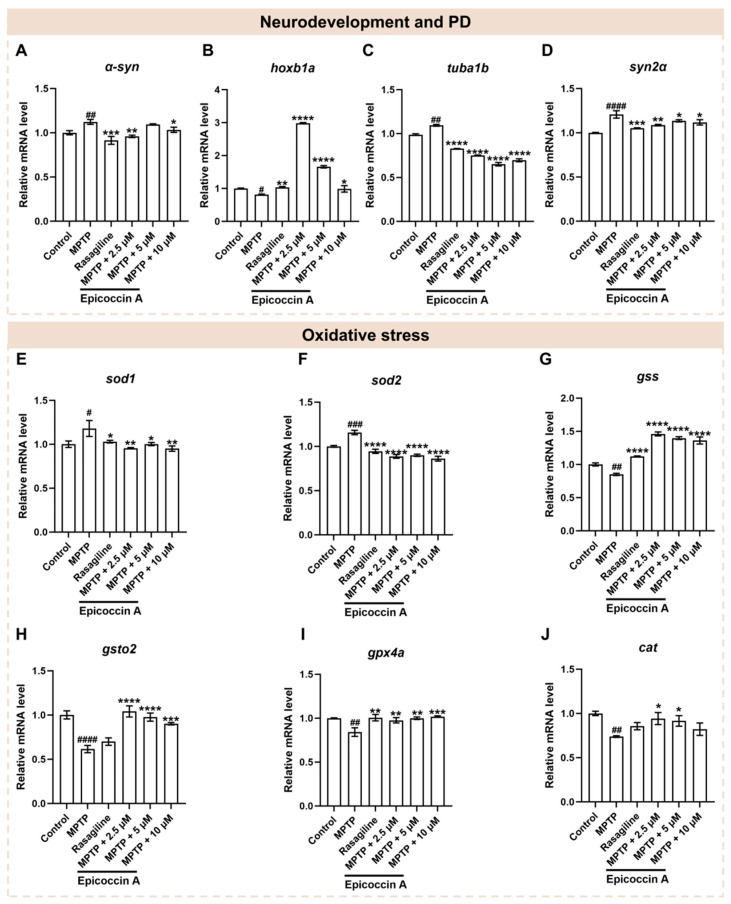
The mRNA expression levels of genes associated with PD, neurodevelopment, and oxidative stress. The expressions of *α-syn* (**A**), *hoxb1a* (**B**), *tuba1b* (**C**), *syn2α* (**D**), *sod1* (**E**), *sod2* (**F**), *gss* (**G**), *gsto2* (**H**), *gpx4a* (**I**), and *cat* (**J**) after epicoccin A co-treatments. The data are presented as mean ± SEM; ^#^ *p* < 0.05, ^##^ *p* < 0.01, ^###^ *p* < 0.001, and ^####^ *p* < 0.0001 compared to the control group; * *p* < 0.05, ** *p* < 0.01, *** *p* < 0.001, and **** *p* < 0.0001 compared to the MPTP group.

Since neural damage is closely associated with the onset and progression of PD, we further investigated the impact of epicoccin A on the expressions of genes related to neurodevelopment and PD. Following MPTP treatment, the expression level of *α-syn* (Figure 7A), a hallmark gene of PD, was significantly elevated, while epicoccin A plus MPTP co-treatment markedly reversed this elevation. Similarly, there was a significant downregulation of gene encoding rhombomere 4 (*hoxb1a*) in response to MPTP treatment as compared to the control (Figure 7B). In contrast, the downregulated expression of *hoxb1a* was significantly reversed after co-treatment with epicoccin A. Furthermore, MPTP treatment led to a remarkable increase in the expressions of tubulin alpha 1b (*tuba1b*) and synapsin IIa (*syn2α*), which was significantly attenuated by epicoccin A co-treatment (Figure 7C,D). These results collectively suggest that the regulation of neurodevelopmental and PD-related genes may be implicated in the protective effect of epicoccin A against MPTP-induced neural damage.

### 2.7. Effect of Epicoccin A on the Dysregulated Expressions of Genes Related to Oxidative Stress

Oxidative stress, intricately linked to neuronal damage, is well recognized as a key factor in PD pathogenesis [38,39]. Thus, we analyzed the mRNA expressions of genes related to oxidative stress to assess the role of epicoccin A in PD. As a result, a significant increase in the mRNA expression levels of superoxide dismutase 1 (*sod1*) (Figure 7E) and superoxide dismutase 2 (*sod2*) (Figure 7F) was observed in the MPTP-treated group as compared to the control. On the contrary, co-treatments with epicoccin A markedly reversed this elevation. Conversely, MPTP treatment significantly decreased the mRNA expression levels of glutathione synthetase (*gss*) (Figure 7G), glutathione S-transferase omega 2 (*gsto2*) (Figure 7H), glutathione peroxidase 4a (*gpx4a*) (Figure 7I), and catalase (*cat*) (Figure 7J). On the contrary, epicoccin A plus MPTP co-treatment increased the expression levels of these genes, except for *cat* in the 10 μM co-treatments. Collectively, these results suggest that epicoccin A may exert a protective role against oxidative stress in PD by downregulating the expressions of peroxidation-related genes and upregulating the expressions of antioxidant genes.

### 2.8. Effect of Epicoccin A on the Aberrant mRNA Levels of Genes Related to Mitophagy

**Figure 8:**
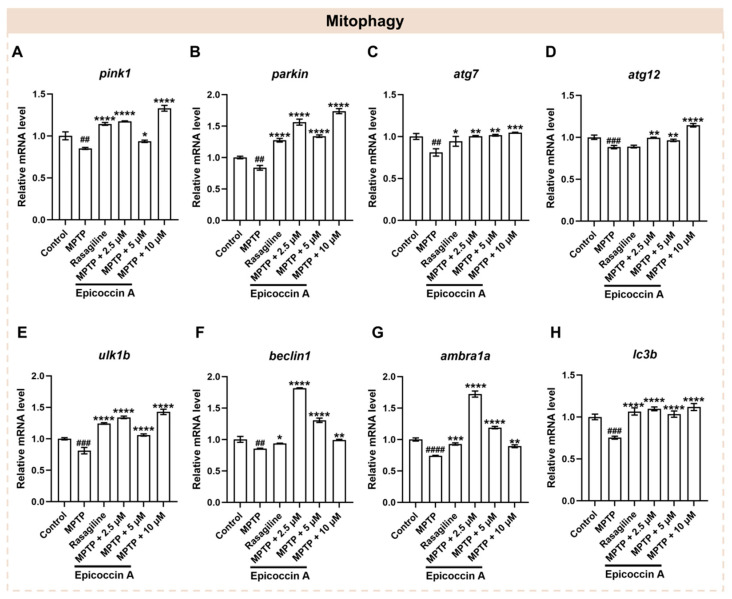
The mRNA expression levels of genes associated with mitophagy. The expressions of *pink1* (**A**), *parkin* (**B**), *atg7* (**C**), *atg12* (**D**), *ulk1b* (**E**), *beclin1* (**F**), *ambra1a* (**G**), and *lc3b* (**H**) after epicoccin A co-treatment. The data are presented as mean ± SEM; ^##^ *p* < 0.01, ^###^ *p* < 0.001, and ^####^ *p* < 0.0001 compared to the control group; * *p* < 0.05, ** *p* < 0.01, *** *p* < 0.001, and **** *p* < 0.0001 compared to the MPTP group.

Mitophagy plays a crucial role in maintaining mitochondrial quality and function, which are closely linked to the onset of PD [33]. To investigate whether epicoccin A co-treatments alleviate PD-like conditions via regulating mitophagy, we assayed the expressions of mitophagy-related genes, including PTEN-induced putative kinase 1 (*pink1*), E3 ubiquitin protein ligase (*parkin*), mitophagy-related gene 7 and 12 (*atg7* and *atg12*), unc-51-like mitophagy activating kinase 1b (*ulk1b*), *beclin1*, activating molecule in beclin1-regulated autophagy (*ambra1a*), and microtubule-associated protein 1 light chain 3B (*lc3b*) [9]. We found that the expression levels of *pink1* and *parkin* (Figure 8A,B) were significantly decreased after MPTP treatment. On the contrary, epicoccin A plus MPTP co-treatment notably reversed the decrease. Further, we found that MPTP treatment significantly downregulated the expression levels of *atg7* and *atg12* genes (Figure 8C,D), while co-treatment with epicoccin A significantly reversed the downregulated expression. Likewise, the mRNA expression levels of *ulk1b*, *beclin1*, and *ambra1a* (Figure 8E–G) were significantly downregulated in the MPTP-treated group, as found in previous studies [40,41]. In contrast, epicoccin A co-treatment markedly prevented the downregulation. Additionally, a significant decrease in the expression level of *lc3b* (Figure 8H) was observed in MPTP treatment, which was reversed by epicoccin A plus MPTP co-treatment. These results suggest that epicoccin A may alleviate PD-like symptoms in zebrafish by activating pink1/parkin-dependent mitophagy.

### 2.9. Interaction Between Epicoccin A and Mitophagy Regulators

**Figure 9:**
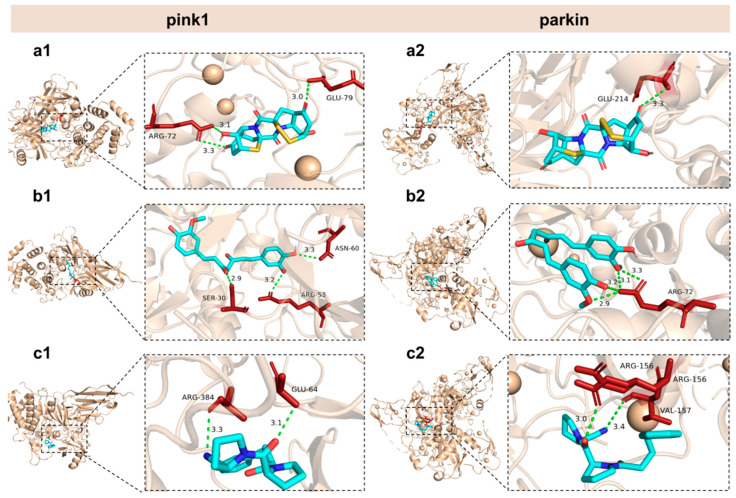
General and local perspectives of docking simulation of interactions between ligands and receptors, with pink1 and parkin being the receptors considered. Epicoccin A (**a1**,**a2**), curcumin (**b1**,**b2**), and KYP-2047 (**c1**,**c2**) were used as molecularly docked ligands. Among them, curcumin and KYP-2047, two well-established potential anti-PD compounds, served as positive controls.

Our study revealed that the aberrant expressions of genes associated with mitophagy were normalized by co-treatments with epicoccin A. Based on these findings, we hypothesized that the observed improvement might be due to potential interactions between epicoccin A and the key molecules involved in mitophagy. Thus, molecular docking analysis was conducted to simulate the interactions and explore the hypothesis. Curcumin and KYP-2047, two well-recognized potential anti-PD compounds, were used as positive controls, with their structures included in Figure S2 of the Supplementary Materials [42]. The results revealed that epicoccin A exhibited stable interactions (binding scores ≤ −7.2 kcal/mol) with all selected regulators involved in pink1/parkin-dependent mitophagy (pink1, parkin, Atg7, Atg12, Ulkl, beclin-1, ambra1, and Lc3b) (Table 1; Figure 9 and Figures S3–S8). Consistent with previous studies, curcumin and KYP-2047, which possess mitophagy-inducing properties, formed relatively stable docking structures with these mitophagy regulators [43]. Noteworthily, epicoccin A stably docked into the binding pockets of all of the tested receptors, exhibiting lower docking energies compared to curcumin and KYP-2047, except for Ulk1 and Lc3b receptors. Specifically, epicoccin A demonstrated the lowest docking energy with parkin, forming three hydrogen bonds and two electrostatic forces with parkin residues Glu79, Arg156, Arg72, and Val157 (Figure S6, Supplementary Material). In addition, the second lowest binding score was observed between epicoccin A and pink1, with the formation of one hydrogen bond and two electrostatic forces when epicoccin A docked with pink1 residues Glu214, Met211, and His68 (Figure S6, Supplementary Material).

### 2.10. Functional Classification and Transcriptome Annotation and Verification

**Figure 10:**
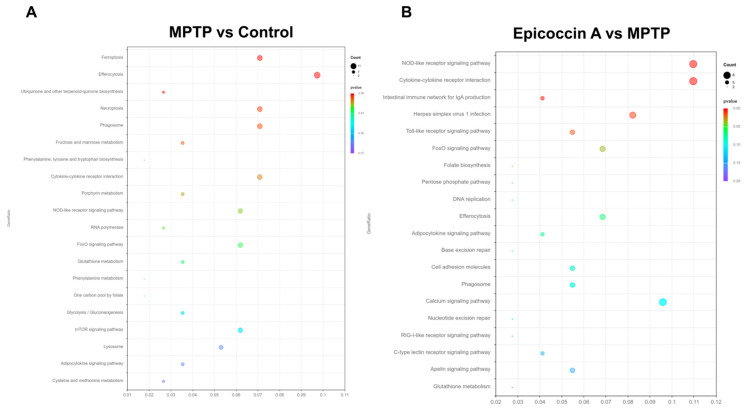
Transcriptome analysis. KEGG enrichment analysis of DEGs in MPTP vs. control (**A**) and epicoccin A vs. MPTP (**B**).

The transcriptome profiles of zebrafish co-treated with epicoccin A at 120 hpf are presented in Figure 10. Hierarchical cluster analysis exhibited distinct clustering patterns, with individuals in the same group forming clustered branches and different groups forming separated branches (Figure S9A, Supplementary Material). Following the screening criteria (*p* < 0.05 and log_2_FC ≥ 1), a total of 540 significant differentially expressed genes (DEGs) were identified in the comparison of MPTP vs. control, including 382 upregulated and 158 downregulated genes (Figure S9B, Supplementary Material). In contrast, when compared to the MPTP group, the epicoccin A group displayed 433 DEGs, comprising 126 upregulated and 307 downregulated genes (Figure S9C, Supplementary Material). Comparative analysis exhibited 124 DEGs were co-expressed in both comparisons, and these DEGs were used for subsequent GO and KEGG enrichment analyses (Figure S9D, Supplementary Material). The GO functional enrichment analysis revealed that DEGs between the control and MPTP-treated groups were annotated to 60 GO categories, including humoral immune response, complement activation, hemoglobin complex, etc. (Figure S9E, Supplementary Material). Similarly, DEGs between the epicoccin A and MPTP treatment were also annotated to 60 GO categories, with primarily enrichment in defense response, leukocyte migration, nuclear membrane, etc. (Figure S9F, Supplementary Material). KEGG enrichment analysis exhibited that common pathways between the MPTP vs. control and epicoccin A vs. MPTP comparisons mainly fell in efferocytosis, the cytokine receptor interaction, the FoxO signaling pathway, the NOD-like signaling pathway, and the adipocytokine signaling pathway (Figure 10A,B). Notably, the FoxO signaling pathway is most closely associated with mitophagy, and is regulated by its upstream regulator, the mTOR signaling pathway. Therefore, we focused on the DEGs within the mTOR/FoxO pathway to further explore the contribution of mitophagy to the protective effect of epicoccin A against PD.

**Figure 11:**
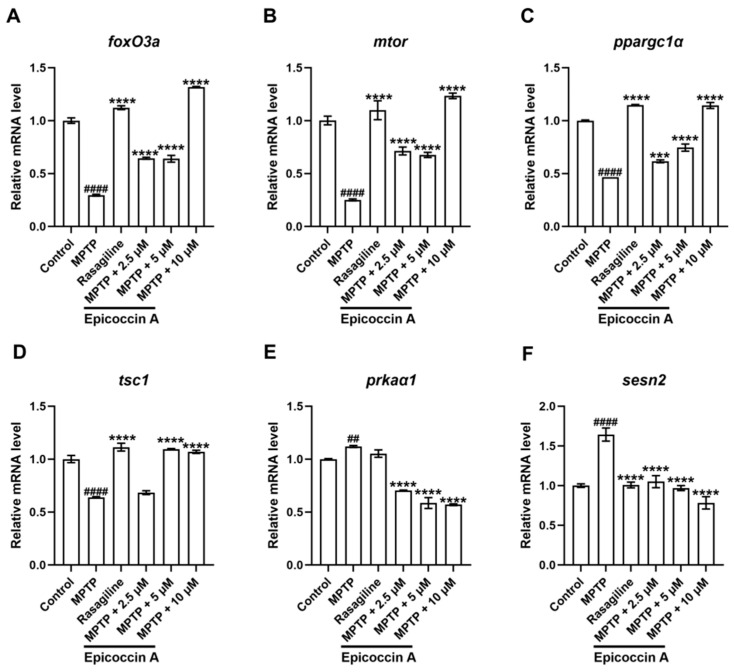
mRNA expression levels of genes related to the mTOR/FoxO signaling pathway. The expressions of *foxO3a* (**A**), *mtor* (**B**), *ppargc1α* (**C**), *tsc1* (**D**), *prkaα1* (homologous gene of *ampk* in zebrafish, (**E**), and *sesn2* (**F**) after epicoccin A co-treatment. The data are presented as mean ± SEM; ^##^ *p* < 0.01 and ^####^ *p* < 0.0001 compared to the control group; *** *p* < 0.001 and **** *p* < 0.0001 compared to the MPTP group.

We validated the expression changes in genes within the mTOR/FoxO signaling pathway. We found that the expression levels of the fork head box O3a (*foxO3a*) (Figure 11A), the mechanistic target of rapamycin kinase (*mtor*) (Figure 11B), the peroxisome proliferator-activated receptor gamma, coactivator 1 alpha (*ppargc1α*) (Figure 11C), and the TSC complex subunit 1A (*tsc1*) (Figure 11D) were significantly decreased after MPTP treatment. Epicoccin A plus MPTP co-treatments reversed this decrease, except for *tsc1* in the co-treatment at 2.5 μM concentration. Conversely, there were significant upregulations in the expression levels of AMP-activated protein kinase (*ampk*, identified as *prkaα1* in zebrafish) (Figure 11E) and sestrin 2 (*sesn2*) (Figure 11F) in the MPTP treatment in comparison to the control. Contrarily, epicoccin A plus MPTP co-treatments remarkably reversed the upregulations in a concentration-dependent manner.

## 3. Discussion

The complexity of pathogenetic factors in PD poses significant challenges for developing effective therapies. Significantly, the identification of numerous pivotal molecular events has paved the way for exploring alternative therapeutic strategies for PD, which may extend conventional treatments such as L-dopa and dopamine agonists. These events include aberrant accumulation and aggregation of α-synuclein, elevated oxidative stress, mitochondrial dysfunction, and enhanced neuroinflammatory response, all of which interact reciprocally with the degeneration of DA neurons and thereby exacerbate the disease progression [44,45,46]. Marine natural products, which exhibit a wide range of bioactivities including mitophagy-activating, antioxidant, and anti-inflammatory properties, are emerging as highly promising candidates for the development of therapies to inhibit the onset and progression of PD [47,48]. For instance, bryostatin, a macrocyclic lactone natural product isolated from the marine organism *Bugula neritina*, exerts neuroprotective effects by activating the Nrf2/HO-1 pathway to reduce oxidative stress [49]. Similarly, Cyclo-(L-Pro-L-Phe), a marine fungus-derived diketopiperazine compound, exerts neuroprotective effects through inhibiting the mitochondrial dysfunction and generation of ROS caused by oxidative stress [24]. Herein, we discovered that epicoccin A exerts the neuroprotective effect on an MPTP-induced PD model in zebrafish, potentially through the enhancement of pink1/parkin-dependent mitophagy and inhibition of excessive oxidative stress.

PD is a neurodegenerative disorder with uncertain pathogenesis, manifested primarily by the degeneration of DA neurons in substantia nigra pars compacta. In this study, we observed a significant loss of DA neurons in the raphe nuclei clusters of zebrafish brains following MPTP exposure, confirming the suitability of MPTP for establishing the PD-like zebrafish model as reported in previous studies [50]. This neural damage was further corroborated by a significant reduction in the expression level of *hoxb1a*, a gene implicated in neurodevelopment, after exposure to MPTP [51,52]. Additionally, *tuba1b*, which is known to be upregulated in the vicinity of injured neuronal cells and damaged axons in CNS, was also significantly elevated following MPTP treatment. Contrarily, epicoccin A co-treatment significantly restored the neuronal damage in PD, as evidenced by the notable increase in the fluorescent-labeled length of DA neurons, remarkable upregulation of *hoxb1a* expression, and significant downregulation of *tuba1b* [53,54,55]. The protective effect of epicoccin A was further substantiated by the significant increase in fluorescent intensity observed in the nervous system of zebrafish brains after co-treatments with epicoccin A, suggesting its potential to promote neuronal recovery and protect the integrity of the nervous system. The disruption of the structure and function of neuronal synapses is a key trigger of neural damage and plays a crucial role in the onset and progression of neurodegenerative diseases, including PD [56]. Co-treatments with epicoccin A significantly reduced PD-associated synaptic injury, as evidenced by the normalization of *syn2α* expression level, which is involved in the synaptogenesis, neuronal differentiation, and neurite formation. Epicoccin A also demonstrated a protective effect against neurovascular damage in PD, suggesting its role in preserving the integrity of the neurovascular unit. Restoring the neurovascular integrity is crucial, as it may help maintain blood–brain barrier function and normal blood supply, which are essential for protecting brains against harmful substances and ensuring normal neuronal function, respectively [57,58].

The progressive degeneration of DA neurons can result in motor impairments through the nigrostriatal pathway [59]. Consistent with previous reports, we observed locomotor disability in zebrafish after MPTP treatment, resembling the characteristic in PD [60]. Co-treatments with epicoccin A significantly improved locomotor deficits in PD-like zebrafish with neuronal loss, as demonstrated by increased traveling speed and total distance. These findings suggest that epicoccin A exerts a therapeutic effect on locomotor impairments associated with PD. Another pathological feature of PD is the formation of LBs, which are primarily composed of aggregated and misfolded α-synuclein [61,62]. Our study showed that the PD-like zebrafish exhibited a marked increase in *α-syn* expression, indicating abnormal accumulation and aggregation of α-synuclein [63]. However, epicoccin A co-treatments significantly decreased the expression level of *α-syn*, suggesting that epicoccin A effectively inhibited α-synuclein aggregation and accumulation, potentially impeding the formation of LBs. The disruption of abnormal α-synuclein aggregation by epicoccin A may reciprocally contribute to the preservation of DA neurons integrity and nervous system function, thereby alleviating locomotor deficits in zebrafish.

Oxidative stress, driven by the accumulation of ROS, can induce mitochondrial dysfunction by disrupting the function of oxidative proteins and impairing membrane potential, thereby triggering further excessive ROS production [64]. These cascades create a vicious loop that ultimately promotes the damage of neuronal cells and neurodegeneration [65]. The regulation of the oxidative stress process holds the potential to restore mitophagy homeostasis and represents a promising therapeutic strategy for mitigating the damage of neuronal cells associated with PD [66]. Consistent with observations in animal models of PD [49], we found an elevated level of ROS in PD-like zebrafish, as shown by a significant increase in fluorescent intensity in the brains of MPTP-treated zebrafish. ROS are highly reactive and can oxidize intracellular biomacromolecules such as proteins, resulting in oxidative damage [67]. Indeed, we detected a significant upregulation of peroxidative genes and downregulation of antioxidant genes in PD-like zebrafish, which may facilitate the oxidative stress process. These alterations could be reversed following epicoccin A co-treatments, with a notable decrease in ROS production in zebrafish brains and the normalization of peroxidative and antioxidant gene expression. These findings suggest epicoccin A may effectively inhibit excessive oxidative stress, disrupting the peroxidation process and restoring antioxidant system efficacy. Given that mitochondria are the major producer of ROS and are highly vulnerable to oxidative stress, the observed decrease in ROS level likely indicates the improvement of mitochondrial function, which ensures a normal supply of energy to neural cells, ultimately facilitating the alleviation of PD symptoms [68].

Mitophagy plays a crucial role in maintaining mitochondrial function and homeostasis, and its disruption leads to the accumulation of dysfunctional mitochondria, culminating in neuronal demise and neurodegenerative disease progression [69]. Numerous studies have shown that MPTP treatment, both in vitro and in vivo, can induce change in mitochondrial membrane potential, leading to mitochondrial dysfunction [70]. Our study also provided evidence that MPTP impairs mitophagy function, as shown by the decreased expressions of *pink1* and *parkin*, two crucial regulators of mitophagy. Disruption of the pink1/parkin-mediated pathway results in the accumulation of damaged mitochondria, which exacerbates mitochondrial dysfunction and contributes to the damage of DA neurons and CNS [71]. Conversely, epicoccin A co-treatments may efficiently recover the mitophagy function, significantly upregulating the expressions of *pink1* and *parkin*, and robustly docking to these two proteins through hydrogen bonding and electrostatic forces. These results suggest epicoccin A restored the function of pink1 and parkin, promoting their synergistical work to orchestrate the degradation of damaged mitochondria through mitophagy [72]. Activation of the ulk complex initiates autophagosome formation and phosphorylates its downstream protein beclin-1 to advance mitophagy process [73]. Epicoccin A co-treatments may promote autophagosome formation by significantly upregulating the expression of the *ulk1b* gene. Epicoccin A notably elevated *beclin1* and *ambra1a* expressions, potentially enhancing the phosphorylation of beclin-1 and its interaction with ambra1a for autophagosome precursor assembly. Additionally, epicoccin A co-treatments appeared to enhance the proper formation of mature autophagic vesicles and restore autophagic function to homeostasis, as indicated by the significant reversal of downregulated atg complex-encoded gene expressions in PD (*atg7* and *atg12*) [74]. Furthermore, the expression of *lc3b*, which encodes a protein located on autophagosome membrane, was significantly upregulated by epicoccin A, indicating its role in mediating the fusion of autophagosomes with lysosomes and subsequent degradation process [75]. In addition, the stable binding of epicoccin A with these mitophagy regulators is consistent with that observed for curcumin and KYP-2047, both of which are well-recognized for their ability to activate mitophagy to alleviate PD [76]. Overall, these results support our hypothesis that epicoccin A may exert its anti-PD effects by activating pink1/parkin-dependent mitophagy.

Based on transcriptomic analysis via KEGG pathway enrichment, we identified six DEGs in the mitophagy-related mTOR/FoxO signaling pathway. These six DEGs were verified to be reversed following epicoccin A co-treatments, as compared to those observed in MPTP treatment. mTOR regulates the initiation of mitophagy by sensing changes in the oxidative stress process, while its downstream FoxO signaling pathway promotes mitophagy by regulating genes involved in mitochondrial quality control and stress response [77]. Regulation of the mTOR/FoxO signaling pathway by epicoccin A ensures the activation of mitophagy under conditions such as oxidative stress or mitochondrial damage, thereby maintaining cellular homeostasis [78]. FoxO3, a key factor in the FoxO pathway, is regulated by a dynamic balance of phosphorylation and deacetylation [79]. High expression levels of sesn2 and ampk will lead to the transition of foxO3 from deacetylation to phosphorylation, thereby inhibiting its transcriptional activity and the expressions of downstream factors, such as pink1 and parkin, ultimately impairing mitophagy [80,81]. As expected, we observed the upregulated expression levels of *sens2* and *prkaα1* genes in zebrafish following MPTP treatment, which suggest the suppression of foxO3 activity. However, co-treatments with epicoccin A significantly reversed this upregulation, promoting the transition of foxO3 from phosphorylation to deacetylation and its activity activation, leading to mitophagy enhancement. Additionally, epicoccin A co-treatments could activate ppargc1α to further enhance the deacetylation process of foxO3 [82,83], as evidenced by the upregulated expression of *ppargc1α* following epicoccin A co-treatments. These results suggest that epicoccin A may activate pink1/parkin-dependent mitophagy by reversing the aberrant gene expression in the upstream mTOR/FoxO signaling pathway of pink1/parkin.

**Figure 12:**
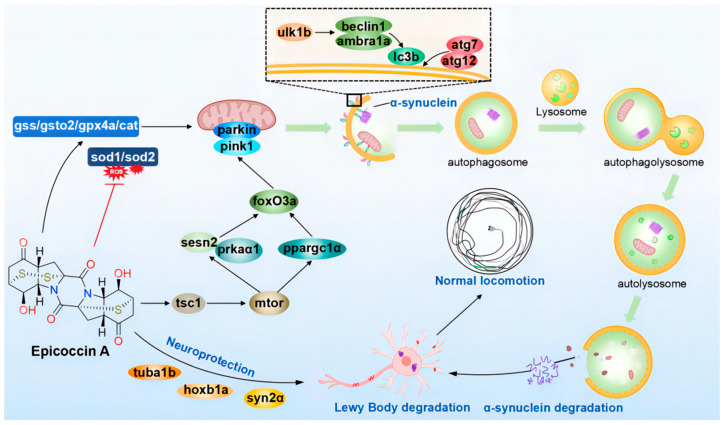
The proposed mechanism underlying the anti-PD effect of epicoccin A. Epicoccin A co-treatments can reverse the abnormal expressions of genes related to neuronal development, contributing to the improvement of neuronal damage in PD. Co-treatments with epicoccin A improved the aberrant gene expressions in the mTOR/FoxO signaling pathway, which might activate pink1/parkin-dependent mitophagy. This process facilitated the degradation of damaged mitochondria and α-synuclein fibrils, thereby inhibiting the formation of LBs. Moreover, epicoccin A co-treatments can inhibit oxidative stress by reducing ROS accumulation, further enhancing mitophagy and consequently alleviating the onset and development of PD.

In summary, our investigation indicated that epicoccin A exerts anti-PD activity mainly by alleviating the loss of DA neurons and neural vasculature, restoring the injury of the nervous system, inhibiting locomotor impairment, and reversing the abnormal expressions of genes related to PD and neural development. Furthermore, epicoccin A could reverse the aberrant gene expressions in the pink1/parkin and mTOR/FoxO signaling pathways as well as in the oxidative stress process. This finding suggests epicoccin A may alleviate PD-like symptoms by activating pink1/parkin-dependent mitophagy and inhibiting excessive oxidative stress. Therefore, epicoccin A may serve as a promising therapeutic option for PD, providing a potential strategy to address the limitation of current PD treatments (Figure 12).

## 4. Materials and Methods

### 4.1. Chemicals and Reagents

The MPTP, phenylthiourea, and tricaine (used as an anesthetic) were purchased from Sigma-Aldrich (St Louis, MO, USA). Rasagiline was purchased from Shanghai Yuanye Bio-Technology Co., Ltd. (Shanghai, China). The rest of chemicals and reagents used in this study were of analytical grade.

### 4.2. Fermentation, Extraction, and Isolation of Epicoccin A

Epicoccin A was extracted from an *Exserohilum* sp. M1–6 [84]. In brief, *Exserohilum* sp. M1-6 were inoculated in 1000 mL conical flasks containing 300 mL liquid medium (glucose 0.1 g/L, maltose 0.2 g/L, yeast extract 0.03 g/L, monosodium glutamate 0.1 g/L, mannitol 0.2 g/L, Na_2_SO_4_ 0.07 g/L, and KH_2_PO_4_ 0.005 g/L). The cultures were extracted by EtOAc and concentrated to yield 98.73 g extract. The extracts were further extracted by 90% MeOH–H_2_O and petroleum ether, yielding 67.81 g of MeOH–H_2_O extract. The methanol layer extract was fractionated into 13 fractions (Fr.1–Fr.13) by VLC, eluting with petroleum and ether–EtOAc. Fr.12.10 (3.1 g) were put on an RP-silica gel column with MeOH-H_2_O (*v*/*v* 20:80–100:0) to yield eleven subfractions (Fr.12.10.1–Fr.12.10.11). Fr.12.10.5 was purified by HPLC on an ODS column (30% MeOH/H_2_O) to yield compound epicoccin A (42.9 mg, t_R_ 12.15 min). Epicoccin A is a colorless powder, and was isolated and analyzed as follows: semi-preparative HPLC was performed on a Waters 1525 system using a semi-preparative C18 (5 µm, 10 × 250 mm, 4 mL/min Cosmosil, Kyoto, Japan) column coupled with a Varian 330 detector. Analytical HPLC was performed on a Waters 1525 system using a semi-preparative C18 (5 µm, 4.6 ID × 250 mm, 1 mL/min Cosmosil, Kyoto, Japan) column coupled with a HPLC Shimadzu LC-10AD VP instrument. The optical rotation was measured on SGWzz-1 digital polarimeter, The NMR spectra were obtained on a Bruker AM-400 spectrometer using TMS as an internal standard or residual solvent signal for referencing (DMSO-*d*_6_ *δ*_H/C_ 2.50/39.52). The optical rotation data for epicoccin A is [α]^D^ + 179 (*c* 0.06, CH_3_OH). The structure of epicoccin A was determined by its ^1^H and ^13^C NMR spectroscopic data (Figure S10, Supplementary Material), which was consistent with the previous studies [23].

### 4.3. Animals

All the experimental protocols were performed aligning with the guidelines of the Animal Care and Ethics Committee of the Biology Institute, Qilu University of Technology (Shandong Academy of Sciences). Zebrafish of wild-type AB strain and transgenic lines (*slc18a2:GFP* and *fli1:GFP*) were acquired from the Biology Institute, Qilu University of Technology (Shandong Academy of Sciences), and transgenic zebrafish (*elavl3:EGFP*) were obtained from the China Zebrafish Resource Center. All these lines were maintained according to the standard protocol [85]. Female and male zebrafish were separately maintained under a 14 h light/10 h dark cycle at 28 ± 0.5 °C and fed with granulated baits and *Artemia salina* (brine shrimp) regularly. The healthy and sexually mature zebrafish were placed in a breeding tank at a male-to-female ratio of 2:2 for fertilization. Zygotes were obtained at 9:00–9:30 a.m. after natural mating the following day, washed, and kept in bathing medium (5 mM NaCl, 0.17 mM KCl, 0.33 mM CaCl_2_, and 0.33 mM MgSO_4_) with the addition of 0.1% methylene blue as a disinfectant. The bathing medium was maintained at 7.1 pH and changed daily. Embryos at 24 hpf were selected under a dissecting microscope (Olympus, Tokyo, Japan) and those normally developed were used for further experimentations.

### 4.4. MPTP and Epicoccin A Treatments

**Figure 13:**
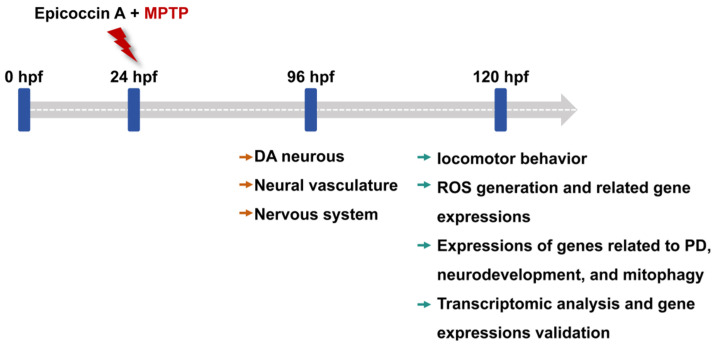
The experimental workflow chart. Larvae at 24 hpf were co-treated with MPTP and each of three different concentrations of epicoccin A from 24 hpf to either 96 hpf or 120 hpf. The developmental assessments of DA neurons, the nervous system, and the neural vasculature were conducted at 96 hpf. At 120 hpf, treated zebrafish were subjected to evaluations of locomotor behavior, ROS generation, expressions of genes related to PD, neurodevelopment, mitophagy, and oxidative stress, as well as transcriptome analysis.

Zebrafish embryos of each strain at 24 hpf were dechorionated manually and transferred randomly to 6-well cell culture plates, with 20 embryos per well in 5 mL bathing medium. To investigate the anti-PD activity of epicoccin A, zebrafish larvae in the wells were divided into 6 groups: control, MPTP, as well as three epicoccin A plus MPTP co-treatments. In addition, rasagiline was used as a positive control to assess the efficacy of epicoccin A, as rasagiline is a common therapeutic drug for PD patients in clinical settings and has demonstrated neuroprotective effects against PD models in vivo and in vitro [86,87]. Zebrafish larvae were then exposed to 60 µM MPTP to induce PD-like symptoms, as shown by previous studies [28]. Epicoccin A was initially dissolved in dimethyl sulfoxide (DMSO) and subsequently diluted with the bathing medium to prepare various working solutions for treating zebrafish. Since no adverse effects on zebrafish morphology were observed in only epicoccin A treatment below 30 μM during the initial toxicity assessment (Supplementary Figure S1), we chose concentrations of epicoccin A below 30 μM for co-treatments. The highest concentration of epicoccin A was set at 10 μM, ensuring the DMSO content did not exceed 0.1% (*v*/*v*). The DMSO concentration was also taken into account in the control group, where 0.1% (*v*/*v*) DMSO was used as the solvent. The positive control group was co-treated with 1 μM rasagiline plus MPTP, both of which were added simultaneously to the bathing medium. Similarly, epicoccin A and MPTP were added into the bathing medium at the same time in the MPTP and epicoccin A co-treatment groups. After treatment, the culture plates were incubated at 28 ± 0.5 °C, and the treated mediums were replaced every 24 h. For the transgenic zebrafish, 0.03 mg/mL phenylthiourea was added to their bathing medium from 6 hpf, so as to inhibit melanin formation and facilitate subsequent observation under a fluorescent microscope. The changes in DA neurons, the nervous system, and the neural vasculature in zebrafish after treatment were evaluated at 96 hpf. Zebrafish at 120 hpf after treatment were used for analyses of locomotor activity, reactive oxygen species (ROS) generation, and gene expressions. The experimental workflow and group division were shown in Figure 13 and Table S1.

### 4.5. Detection of Length of DA Neurons and Fluorescent Intensity of Nervous System

Fluorescent-labeled *Tg* (*elavl3:EGFP*) and *Tg* (*slc18a2:GFP*) zebrafish were used to evaluate the effect of epicoccin A on DA neurons and the nervous system in PD-like zebrafish, respectively. Zebrafish at 96 hpf were anesthetized and 8 individuals were randomly selected from each group for visual observation and image acquisition in a fluorescent microscope (Zeiss, Jena, Germany) or confocal microscope (Olympus, Tokyo, Japan). We measured the length of DA neurons and quantified the fluorescent intensity of the nervous system in zebrafish brains, as implemented in Image Pro Plus v.5.1 software (Media Cybernetics, MD, USA).

### 4.6. Assessment of Cerebral Vascular Development

Cerebral vasculatures in zebrafish brains were observed and assessed as previously described [88]. *Tg* (*flk1:EGFP*) zebrafish with developing vascular endothelium labeled by green fluorescent protein were collected at 96 hpf following treatment. Images of 8 zebrafish larvae from each group were acquired using confocal microscope. We compared the alteration of microvessels in zebrafish brains at dorsal view to evaluate the effect of epicoccin A on the neural vasculature of the zebrafish PD model.

### 4.7. Detection of ROS Generation in Zebrafish Larvae

ROS levels in zebrafish larvae were detected using the ROS assay kit (Beyotime Biotechnology, Shanghai, China) according to the protocol recommended by the manufacturer. Zebrafish larvae at 120 hpf after treatment were transferred to a 24-well plate, and exposed to 30 µM DCFH-DA solution and maintained at 28 ± 0.5 °C for 40 min. Detection of ROS generation in zebrafish larvae (n = 8) were then carried out using a fluorescent microscope (Olympus, Tokyo, Japan).

### 4.8. Behavioral Testing

To comprehensively assess the effect of epicoccin A on PD-like locomotion profiles of zebrafish, behavioral assays were performed. We conducted the behavioral recording in a soundproof room between 10:00 a.m. and 5:00 p.m. Zebrafish larvae at 120 hpf from each group were distributed into 48-well plates, with one individual per well in 1 mL bathing medium. The 48-well plate was placed in a black box of the Zebralab system (Viewpoint, Lyon, France) to record the trajectory. After a 10 min acclimation period, zebrafish locomotion was tracked for 20 min, with the LUX value set to 0%. Zebralab software v3.3 was used to analyze the digital tracks of each zebrafish. Twenty-two zebrafish larvae per group were used for the calculation of total distances and average speed.

### 4.9. RNA Extraction and Real-Time Quantitative PCR (RT-qPCR)

Following treatment, the brains of zebrafish larvae (n = 30 per group) at 120 hpf were dissected and homogenized for RNA extraction. Total RNA was extracted using the EASY spin plus RNA mini kit (Aidlab Biotechnologies, Beijing, China) according to the instructions of manufacturer. cDNA was synthesized using the NovoScript^®^ Plus all-in-one 1 st strand cDNA synthesis supermix (Novoprotein, Shanghai, China). RT-qPCR was performed using NovoStart^®^ SYBR qPCR supermix plus (Novoprotein, Shanghai, China) on a Light Cycler^®^ 96 System (Roche, Switzerland). The conditions for RT-qPCR amplification were as follows: pre-denaturation at 95 °C for 180 s, followed by 40 cycles of denaturation at 95 °C for 15 s and annealing and extension at 60 °C for 30 s, and finally a melting curve amplification including 95 °C for 15 s, 65 °C for 60 s, and 95 °C for 1 s. Runs were performed in triplicate and *rpl13a* is used as a housekeeping gene to normalize the mRNA levels of genes related to the hallmark of PD (*α-syn*), neurodevelopment (*hoxb1a*, *tuba1b*, and *syn2α*,), oxidative stress (*sod1*, *sod2*, *gss*, *gsto2*, *gpx4a*, and *cat*), pink1/parkin-dependent mitophagy (*pink1*, *parkin*, *atg7*, *atg12*, *ulk1b*, *beclin1*, *ambra1a*, and *lc3b*), and the mTOR/FoxO signaling pathway (*foxO3a*, *mtor*, *ppargc1α*, *tsc1*, *prkaα1*, and *sesn2*).

### 4.10. Molecular Docking

Eight key molecules in mitophagy, identified through gene expressions, including pink1, parkin, Atg7, Atg12, Ulk1, beclin-1, ambra1, and Lc3b, were used as docking ligands. The crystal structures of the pink1–ubiquitin complex (6EQI), E3 ubiquitin protein ligase (5C1Z), Atg7–Atg3 complex (3T7G), Atg5–Atg16 complex (4GDL), Ulk1–hesperadin complex (6QAS), Beclin1–gabarapl1 complex (6HOI), activating molecule in beclin-1-regulated autophagy protein 1 (8WQR), and NLIR–Lc3b complex (5XAD) were obtained from the Protein Data Bank https://www.rcsb.org/ (accessed on 10 December 2024). Molecular docking simulations were conducted using epicoccin A and two established therapeutic agents, curcumin and KYP-2047, as ligands in the context of PD. Molecular docking analysis of receptors and ligands was performed in AutoDock v1.2.5, following the methodology described in the previous studies [89]. Before docking, the crystal structures of target proteins were optimized in AutoDock through a series of steps, including removing interfering molecules and water molecules, cleaning the proteins, adding hydrogen atoms, and applying a force field, to ensure proper molecular interactions. Additionally, the structure of epicoccin A was minimized using AutoDock and then exported in a PDBQT format. Subsequently, automated molecular docking was conducted utilizing the AutoDock Vina and visualized with PyMol 2.5 software. The center coordinates (center x/y/z) and box dimensions (size x/y/z) were set according to the parameters delineated in Table 1. The conformation with the lowest binding scores was considered as the optimal docking conformation and used for evaluation.

### 4.11. Transcriptome Analysis

Following the treatment described in Section 4.4, zebrafish larvae from three groups, i.e., control, MPTP, and epicoccin A (10 µM) plus MPTP, were collected for transcriptome profiling to explore the underlying mechanisms. Each group was analyzed in quadruplicate with 50 larvae per replicate. The transcriptome sequencing and analysis were performed by Novogene Co., Ltd. (Beijing, China). DESeq2 v.1.20.0 (Novogene Co., Ltd., Tianjin, China) was used to perform differential expression analysis between the control, MPTP, and epicoccin A. DEGs with an adjusted *p*-value ≤ 0.05 and absolute fold change ≥ 2 were identified by DESeq2, using the negative binomial distribution method. The phyper function in R was used to perform GO and KEGG pathway enrichment analysis for DEGs. GO terms and KEGG pathways were considered significantly enriched when the adjusted *p*-value ≤ 0.05. Volcano plots were generated to visualize the distribution of DEGs based on log_2_FC ≥ 1 on the *x*-axis and *p <* 0.05 on the *y*-axis.

### 4.12. Statistical Analysis

GraphPad Prism v.8.0 (GraphPad Software; San Diego, CA, USA) were used to analyze the result data by one-way ANOVA followed by Dunnett’s multiple comparison. All data were expressed as mean ± SEM, with *p* < 0.05 considered as statistically significant.

## Figures and Tables

**Table 1:** Binding score, binding site, and docking region size for the optimal conformation of epicoccin A docked with mitophagy regulators.

Receptor	Ligand	Binding Score (kcal/mol)	Binding Site (X, Y, Z)	Docking Region Size (X, Y, Z)
pink1	epicoccin A	−8.8	60.658, 7.027, 18.506	94.85 Å, 72.26 Å, 73.77 Å
	curcumin	−8.4		
	KYP-2047	−8.0		
parkin	epicoccin A	−9.9	−20.445, 3.618, 25.773	111.65 Å, 101.01 Å, 111.65 Å
	curcumin	−8.0		
	KYP-2047	−8.4		
Atg7	epicoccin A	−8.3	18.923, −50.614, 19.942	94.15 Å, 70.23 Å, 94.15 Å
	curcumin	−7.3		
	KYP-2047	−7.0		
Atg12	epicoccin A	−8.7	155.126, 4.886, 19.741	82.25 Å, 82.25 Å, 82.25 Å
	curcumin	−7.1		
	KYP-2047	−7.3		
Ulk1	epicoccin A	−8.2	0.217, 35.817, 40.043	85.75 Å, 74.86 Å, 54.44 Å
	curcumin	−8.3		
	KYP-2047	−9.0		
beclin-1	epicoccin A	−8.6	−4.976, 0.256, −17.797	50.40 Å, 66.15 Å, 48.30 Å
	curcumin	−7.1		
	KYP-2047	−7.2		
ambra1	epicoccin A	−8.4	126.784, 129.570, 130.763	90.21 Å, 90.21 Å, 123.55 Å
	curcumin	−8.3		
	KYP-2047	−8.6		
Lc3b	epicoccin A	−7.2	132.28, 94.936, 136.924	37.66 Å, 118.65 Å, 65.91 Å
	curcumin	−7.7		
	KYP-2047	−7.3		

## Data Availability

The data presented in the current study are available on request from the corresponding author.

## Supplementary Materials

The following supporting information can be downloaded at: https://www.mdpi.com/article/10.3390/md23040175/s1, Table S1. Experimental groups designed for evaluating the neuroprotective effect of epicoccin A on MPTP-induced PD model; Table S2. Sequences of RT-qPCR primers. Forward and reverse primers for the genes related to neurodevelopment, PD, oxidative stress, and mitophagy; Figure S1. Morphological changes of zebrafish at 72, 96, and 120 hpf after epicoccin A treatments. The areas indicated by the red arrows in the figure are the pericardial edema and yolk sac edema regions of the zebrafish, respectively. Larvae without detectable heartbeats were deemed dead. Scale bar, 1 mm for the overall views of all individuals in each well, and 500 μm for the lateral views of individual zebrafish; Figure S2. The two-dimensional (2D) structures of curcumin and KYP-2047; Figure S3. General and local perspectives of docking simulation of interactions between ligands and receptors, with Atg7 and Atg12 being the receptors considered. Epicoccin A (a1 and a2), curcumin (b1 and b2), and KYP-2047 (c1 and c2) were used as molecularly docked ligands; Figure S4. General and local perspectives of docking simulation of interactions between ligands and receptors, with Ulk1 and beclin-1 being the receptors considered. Epicoccin A (a1 and a2), curcumin (b1 and b2), and KYP-2047 (c1 and c2) were used as molecularly docked ligands; Figure S5. General and local perspectives of docking simulation of interactions between ligands and receptors, with ambra1 and Lc3b being the receptors considered. Epicoccin A (a1 and a2), curcumin (b1 and b2), and KYP-2047 (c1 and c2) were used as molecularly docked ligands; Figure S6. Two-dimensional (2D) diagram of the interaction sites between ligands and receptors, with pink1 and parkin being the receptors considered. Epicoccin A (a1 and a2), curcumin (b1 and b2), and KYP-2047 (c1 and c2) were used as molecularly docked ligands; Figure S7. Two-dimensional (2D) diagram of the interaction sites between ligands and receptors, with Atg7, Atg12, and Ulk1 being the receptors considered. Epicoccin A (a1, a2, and a3), curcumin (b1, b2, and b3), and KYP-2047 (c1, c2, and c3) were used as molecularly docked ligands; Figure S8. Two-dimensional (2D) diagram of the interaction sites between ligands and receptors, with beclin-1, ambra1, and Lc3b being the receptors considered. Epicoccin A (a1, a2, and a3), curcumin (b1, b2, and b3), and KYP-2047 (c1, c2, and c3) were used as molecularly docked ligands; Figure S9. Functional classification and annotation of the transcriptome. (A) Heatmap comparisons between the control, MPTP, and epicoccin A groups. The vertical axis represents gene identifiers and the horizontal axis lists the sample names. Red colors indicate upregulated genes, and blue colors indicate downregulated genes. Volcano plots of DEGs in the MPTP group compared with the control group (B), as well as the epicoccin A group compared with the MPTP group (C), with red dots indicating upregulated genes, green dots indicating downregulated genes, and blue dots indicating unchanged genes. (D) Venn diagram of common and specific DGEs between the MPTP vs. control and epicoccin A vs. MPTP comparisons. GO analysis of DEGs involved in MPTP vs. control (E) and epicoccin A vs. MPTP (F); Figure S10. 1H and 13C NMR spectroscopic data of epicoccin A. The 1H NMR (400 MHz, DMSO-d6) spectrum data of epicoccin A: δH 6.20 (1H, br s, 8′-OH), 6.08 (1H, br s, 8-OH), 4.62 (2H, overlapped, H-8, H-9′), 4.49 (1H, br d, J = 8.5 Hz, H-9), 3.99 (1H, br s, H-8′), 3.73 (2H, overlapped, H-7, H-7′), 3.12 (2H, dd, J = 17.0, 11.0 Hz, H-6′), 3.11 (1H, br d, J = 8.5, Hz, H-4), 3.08 (1H, dd, J = 18.0, 12.0 Hz, H-6b), 3.02 (1H, br d, J = 6.8, Hz, H-4′), 2.91 (2H, overlapped, H-3′, H-6′), 2.85 (1H, d, J = 13.0 Hz, H-3b), 2.76 (1H, d, J = 13.0 Hz, H-3a′), 2.58(2H, d, J = 13.0 Hz, H-3), 2.45 (1H, d, J = 18.0 Hz, H-6a); The 13C NMR (100 MHz, CDCl3) spectrum data of epicoccin A: δC 207.9 (C, C-5), 207.6 (C, C-5′), 162.1 (C, C-1), 159.9 (C, C-1′), 146.6 (C, C-4), 74.3 (C, C-2), 70.6 (C, C-2′), 64.9 (CH, C-8), 64.8 (CH, C-8′), 62.4 (CH, C-9), 60.3 (CH, C-9′), 45.7 (CH2, C-3), 45.6 (CH2, C-7), 45.1 (CH, C-4′), 35.7 (CH2, C-4′), 43.5 (CH, C-4), 43.4 (CH2, C-3′), 41.9 (CH, C-7′), 41.4 (CH2, C-6′), 38.1 (CH2, C-6).

## Abbreviations

The following abbreviations are used in this manuscript:

MPTP	1-methyl-4-phenyl-1,2,3,6-tetrahydropyridine
PD	Parkinson’s disease
ROS	Reactive oxygen species
LBs	Lewy bodies
CNS	Central nervous system
hpf	Hours post-fertilization
