# Supplementary material for: Epicoccin A Ameliorates PD-like Symptoms in Zebrafish: Enhancement of PINK1/Parkin-Dependent Mitophagy and Inhibition of Excessive Oxidative Stress
Source: Mar Drugs. 2025 Apr 17;23(4):175. doi: 10.3390/md23040175 (PMC12028493; doi:10.3390/md23040175)
Supplement: Supplementary file 1 [file marinedrugs-23-00175-s001.zip › marinedrugs-3553492-Supplementary.pdf]

Supplementary Table S1. Experimental groups designed for evaluating the neuroprotective effect of epicoccin A on MPTP-induced PD model.

Experimental groups	Mediums for treatment
Control	Bathing medium
MPTP	60 μM MPTP
Rasagiline	1 μM Rasagiline + 60 μM MPTP
Epicoccin A 2.5	2.5 μM Epicoccin A + 60 μM MPTP
Epicoccin A 5	5 μM Epicoccin A + 60 μM MPTP
Epicoccin A 10	10 μM Epicoccin A+ 60 μM MPTP

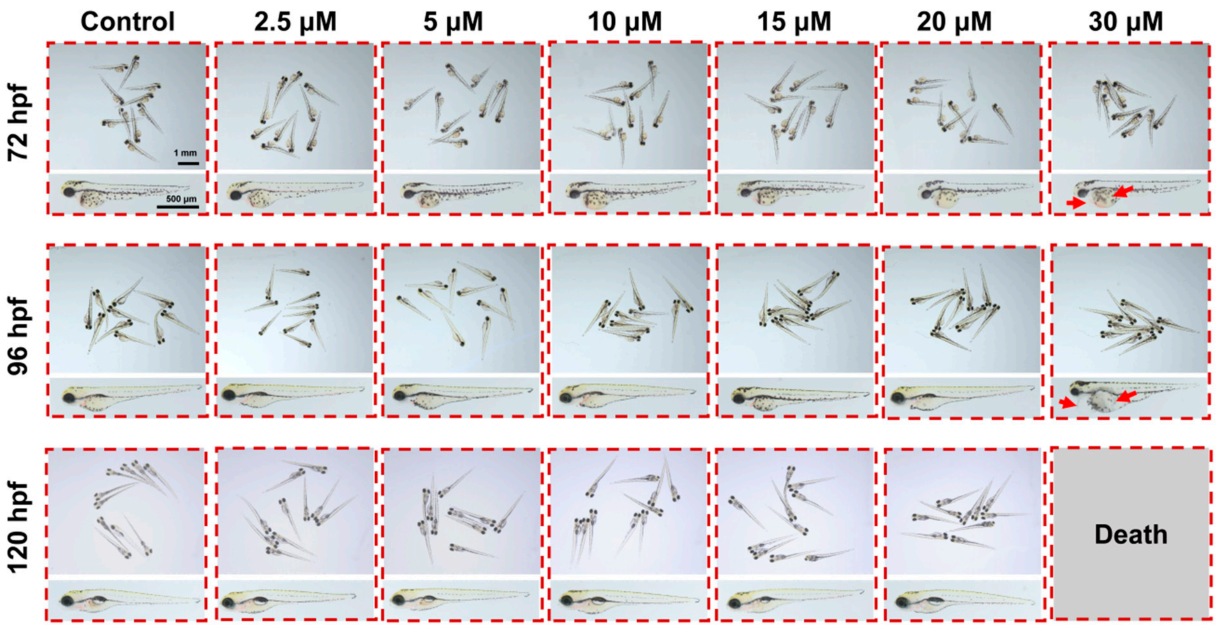

Supplementary Figure S1. Morphological changes of zebrafish at 72, 96, and 120 hpf after epicoccin A treatments. The areas indicated by the red arrows in the figure are the pericardial edema and yolk sac edema regions of the zebrafish, respectively. Larvae without detectable heartbeats were deemed dead. Scale bar, 1 mm for the overall views of all individuals in each well, and 500 μm for the lateral views of individual zebrafish.

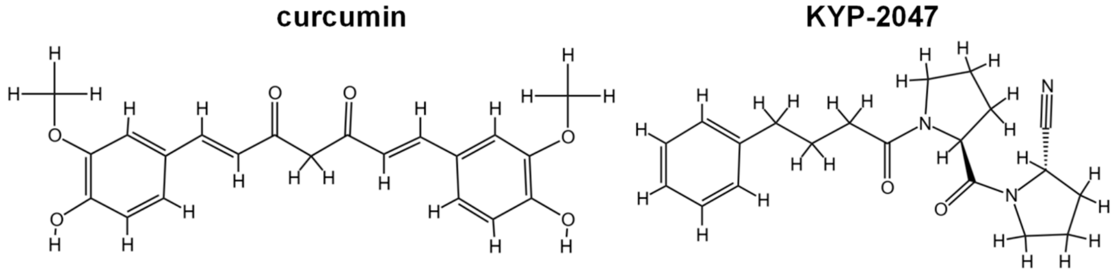

Supplementary Figure S2. The two-dimensional (2D) structures of curcumin and KYP-2047.

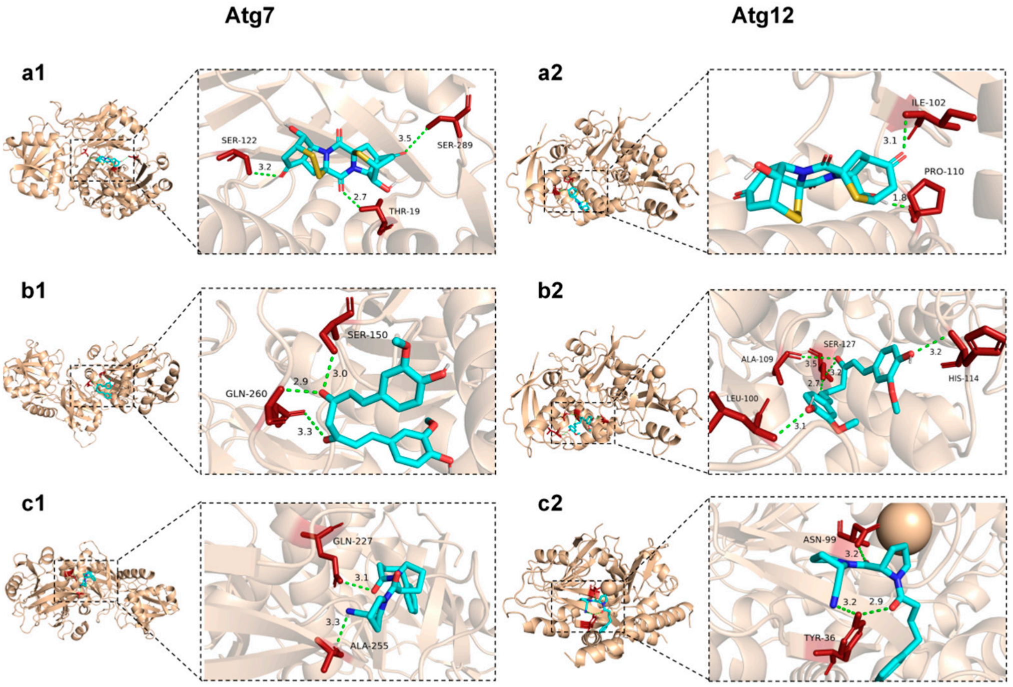

Supplementary Figure S3. General and local perspectives of docking simulation of interactions between ligands and receptors, with Atg7 and Atg12 being the receptors considered. Epicoccin A (a1 and a2), curcumin (b1 and b2), and KYP-2047 (c1 and c2) were used as molecularly docked ligands.

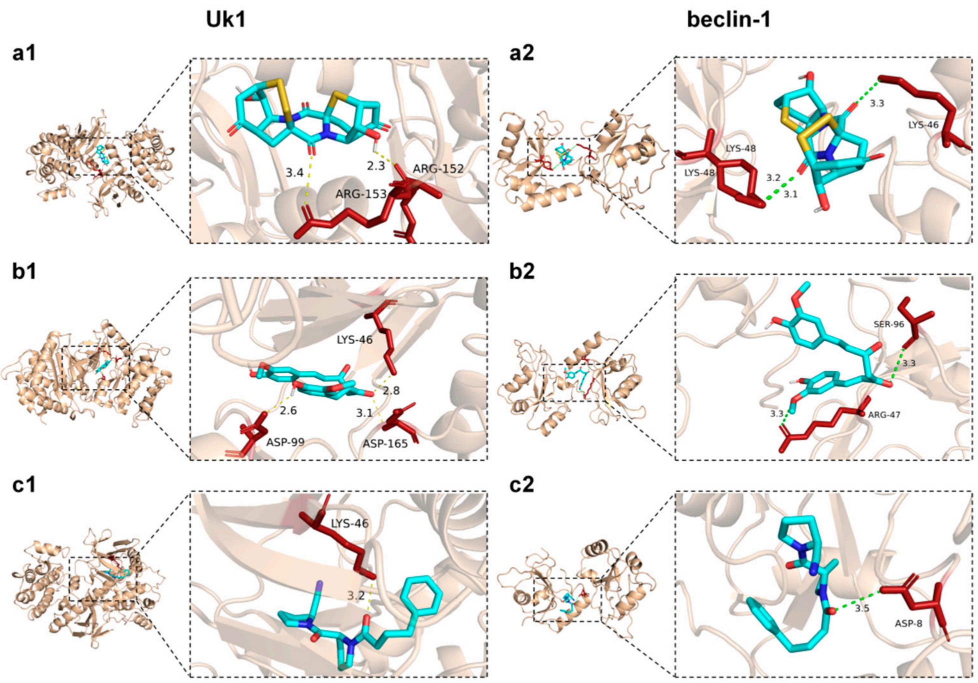

Supplementary Figure S4. General and local perspectives of docking simulation of interactions between ligands and receptors, with Ulk1 and beclin-1 being the receptors considered. Epicoccin A (a1 and a2), curcumin (b1 and b2), and KYP-2047 (c1 and c2) were used as molecularly docked ligands.

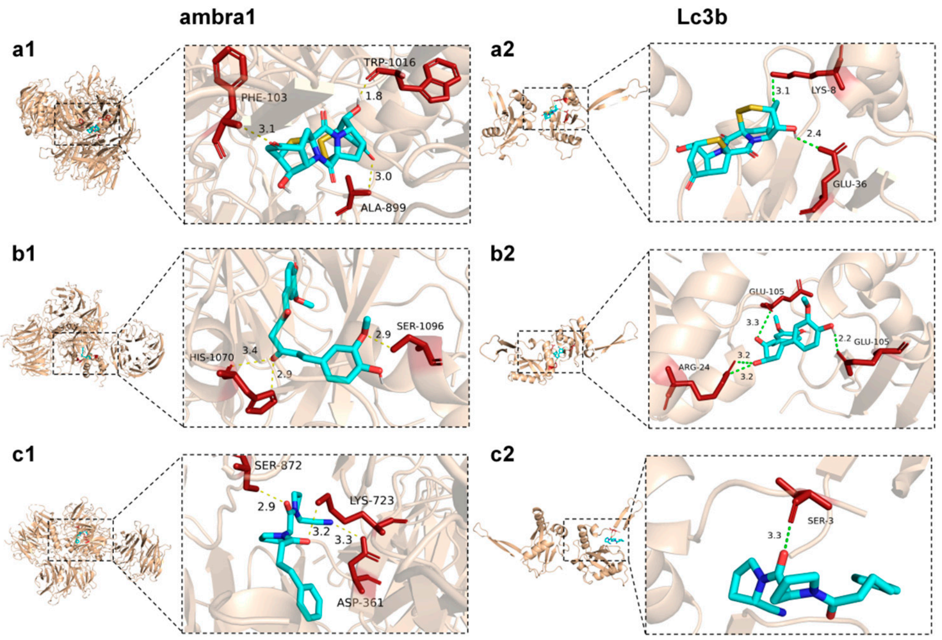

Supplementary Figure S5. General and local perspectives of docking simulation of interactions between ligands and receptors, with ambra1 and Lc3b being the receptors considered. Epicoccin A (a1 and a2), curcumin (b1 and b2), and KYP-2047 (c1 and c2) were used as molecularly docked ligands.

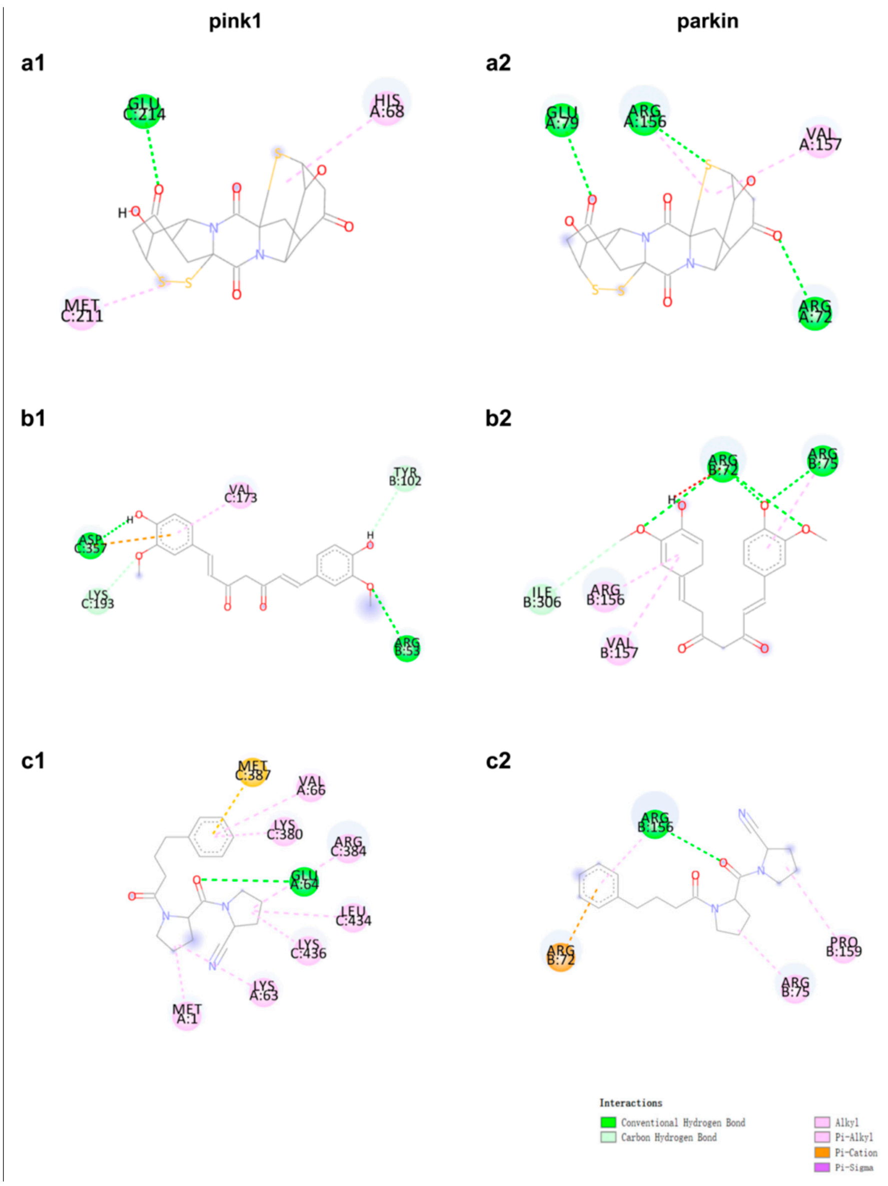

Supplementary Figure S6. Two-dimensional (2D) diagram of the interaction sites between ligands and receptors, with pink1 and parkin being the receptors considered. Epicoccin A (a1 and a2), curcumin (b1 and b2), and KYP-2047 (c1 and c2) were used as molecularly docked ligands.

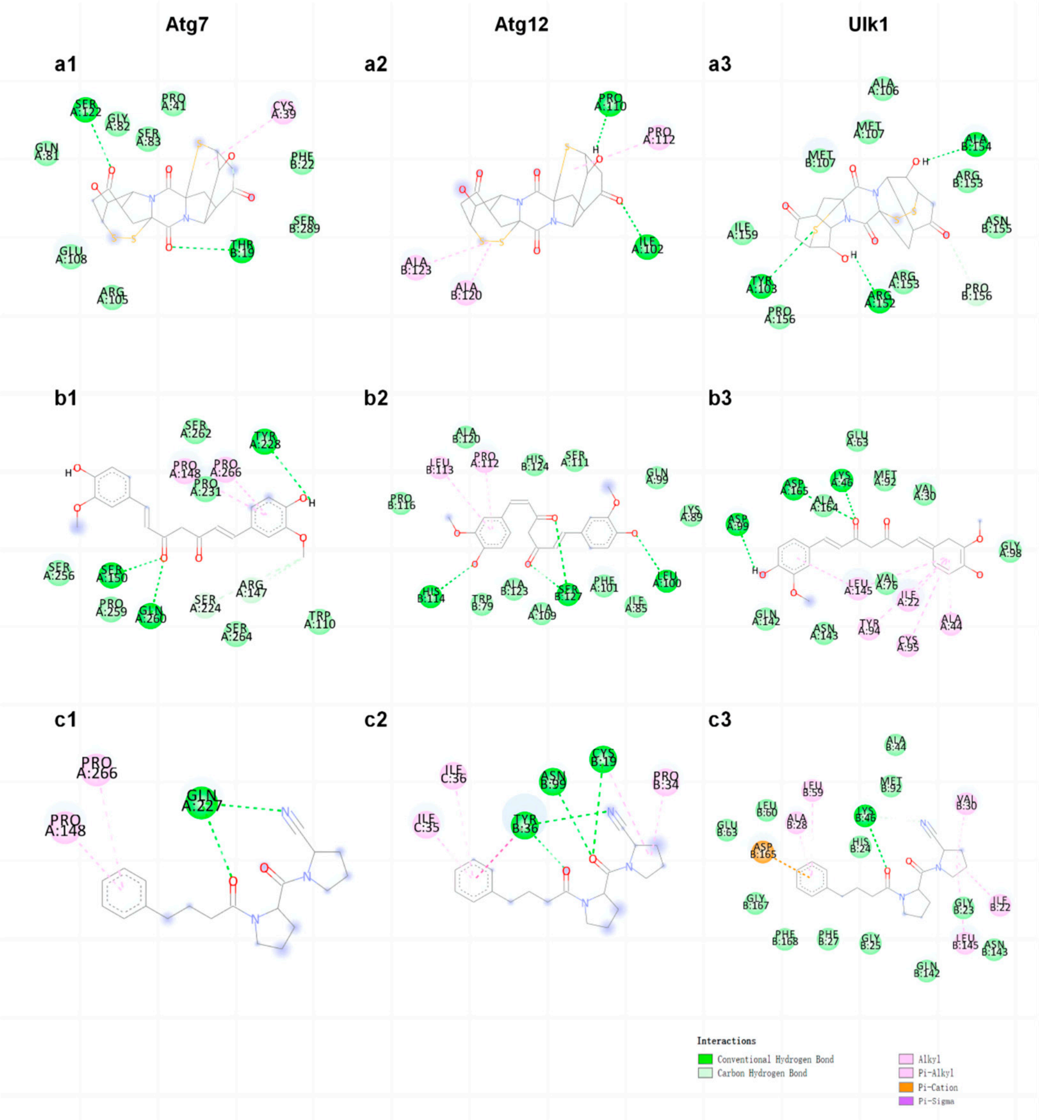

Supplementary Figure S7. Two-dimensional (2D) diagram of the interaction sites between ligands and receptors, with Atg7, Atg12, and Ulk1 being the receptors considered. Epicoccin A (a1, a2, and a3), curcumin (b1, b2, and b3), and KYP-2047 (c1, c2, and c3) were used as molecularly docked ligands.

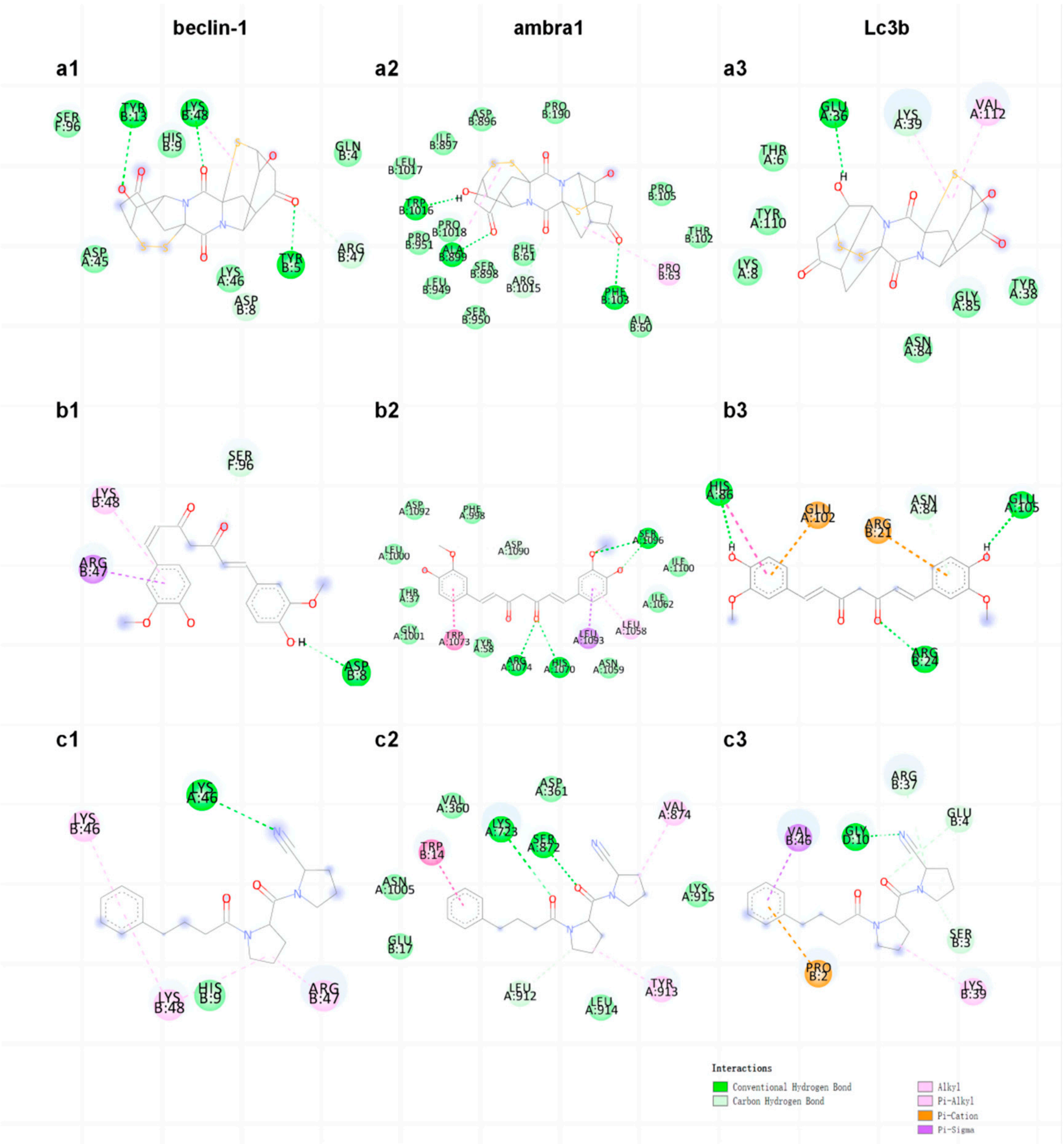

Supplementary Figure S8. Two-dimensional (2D) diagram of the interaction sites between ligands and receptors, with beclin-1, ambra1, and Lc3b being the receptors considered. Epicoccin A (a1, a2, and a3), curcumin (b1, b2, and b3), and KYP-2047 (c1, c2, and c3) were used as molecularly docked ligands.

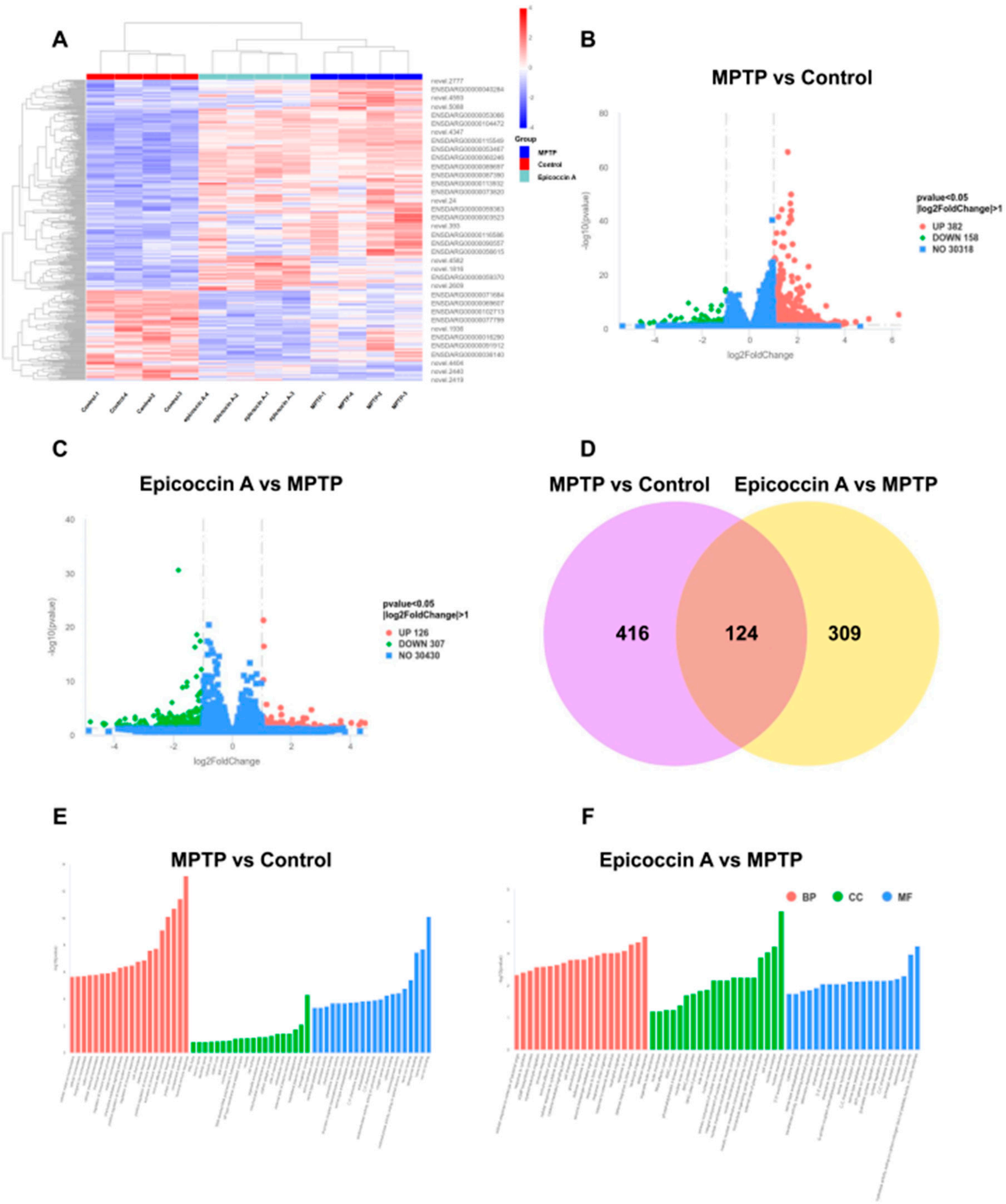

Supplementary Figure S9. Functional classification and annotation of the transcriptome. (A) Heatmap comparisons between the control, MPTP, and epicoccin A groups. The vertical axis represents gene identifiers and the horizontal axis lists the sample names. Red colors indicate upregulated genes, and blue colors indicate downregulated genes. Volcano plots of DEGs in the MPTP group compared with the control group (B), as well as the epicoccin A group compared with the MPTP group (C), with red dots indicating upregulated genes, green dots indicating downregulated genes,

and blue dots indicating unchanged genes. (D) Venn diagram of common and specific DGEs between the MPTP vs Control and epicoccin A vs MPTP comparisons. GO analysis of DEGs involved in MPTP vs Control (E) and epicoccin A vs MPTP (F).

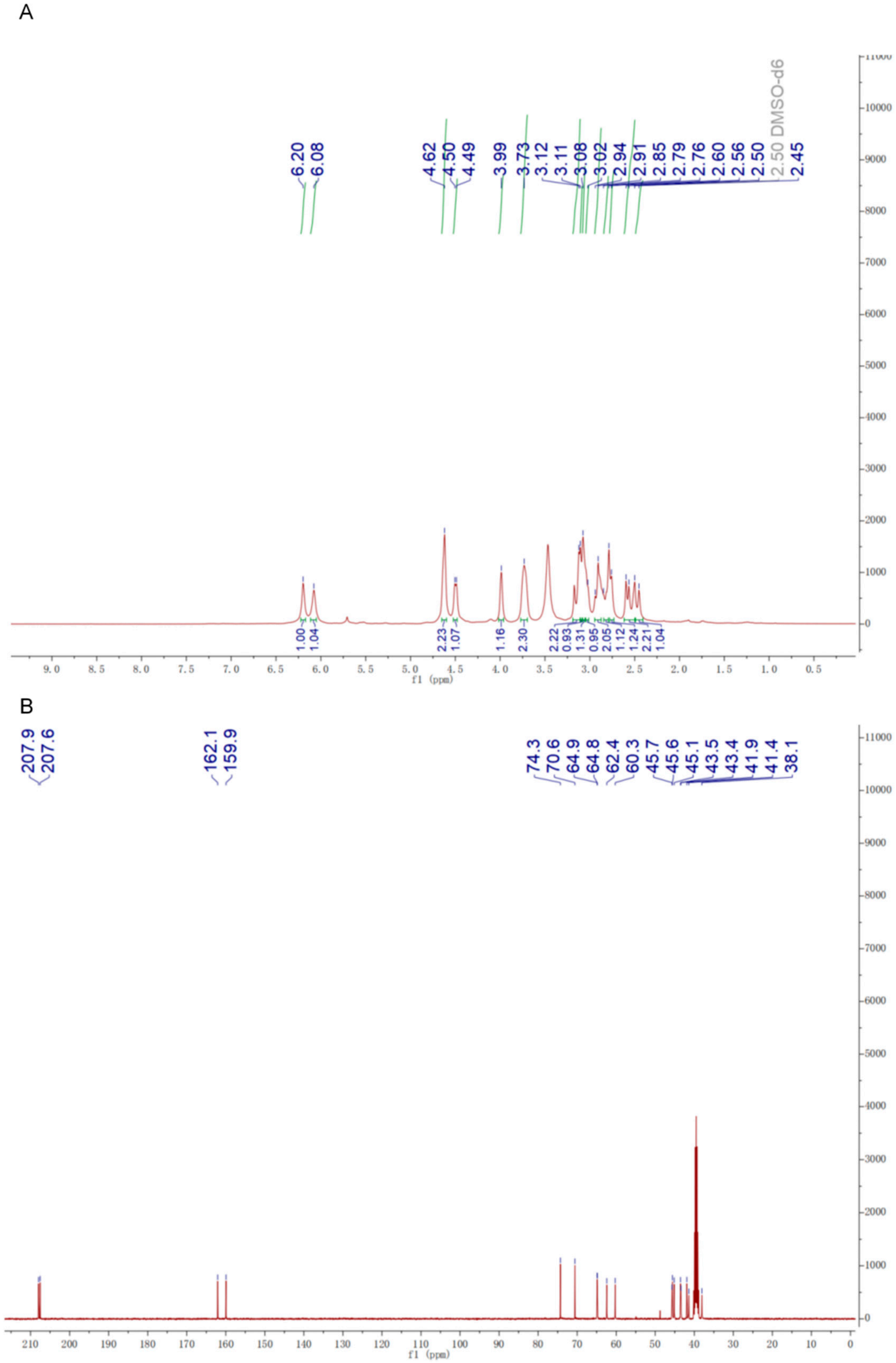

Supplementary Figure S10. ^{1}H and ^{13}C NMR spectroscopic data of epicoccin A. The^{1} H NMR (400 MHz, DMSO-d_6) spectrum data of epicoccin A: δ_H 6.20 (1H, br s, 8'-OH), 6.08 (1H, br s, 8-OH), 4.62 (2H, overlapped, H-8, H-9'), 4.49 (1H, br d, J = 8.5 Hz, H-9), 3.99 (1H, br s, H-8'), 3.73 (2H, overlapped, H-7, H-7'), 3.12 (2H, dd, J = 17.0, 11.0 Hz, H-6'), 3.11 (1H, br d, J = 8.5, Hz, H-4), 3.08 (1H, dd, J = 18.0, 12.0 Hz, H-6b), 3.02 (1H, br d, J = 6.8, Hz, H-4'), 2.91 (2H, overlapped, H-3', H-6'), 2.85 (1H, d, J = 13.0 Hz, H-3b), 2.76 (1H, d, J = 13.0 Hz, H-3a'), 2.58(2H, d, J = 13.0 Hz, H-3), 2.45 (1H, d, J = 18.0 Hz, H-6a); The ^{13}C NMR (100 MHz, $CDCl_3$) spectrum data of epicoccin A: δ_C 207.9 (C, C-5), 207.6 (C, C-5'), 162.1 (C, C-1), 159.9 (C, C-1'), 146.6 (C, C-4), 74.3 (C, C-2), 70.6 (C, C-2'), 64.9 (CH, C-8), 64.8 (CH, C-8'), 62.4 (CH, C-9), 60.3 (CH, C-9'), 45.7 (CH_2, C-3), 45.6 (CH_2, C-7), 45.1 (CH, C-4'), 35.7 (CH_2, C-4'), 43.5 (CH, C-4), 43.4 (CH_2, C-3'), 41.9 (CH, C-7'), 41.4 (CH_2, C-6'), 38.1 (CH_2, C-6).

Supplementary Table S2. Sequences of RT-qPCR primers. Forward and reverse primers for the genes related to neurodevelopment, PD, oxidative stress, and mitophagy.

Gene	Sequence of the forward primer (5'–3')	Sequence of the reverse primer (5'–3')
rpl13a	TCTGGAGGACTGTAAGAGGTATGC	AGACGCACAATCTTGAGAGCAG
α-syn	AGGAAGGCGTGATGTTTGTG	CTGGTTGTCGTCGTATGTCTGT
hoxb1a	CGCTGACTTATCGGCCTCTC	CAAGTGTGGCAGCAATCTCC
tuba1b	AATCACCAATGCTTGCTTCGAGCC	TTCACGTCTTTGGGTACCACG
syn2α	TAATGGGGGAACATCAGGC	GTGGAGCGTTCTTTACTTCG
sod1	AACATGGTTTCCACGTCCAT	CGGTCACATTACCCAGGTCT
sod2	TGTTGGTTGGTCGCTTGTAT	GTGCTTCTGTCTGGAGGTCA

gss	CAGTGAACTGGGTGCATTTGG	GTGGATTGTCCAGCACTGCTACTC
gsto2	ATGGCTTCATCTCCAAAATGC	AGGGCAGAATCTCATGCTGTAG
gpx4a	ATTACGCATCCTGGCTTTC	GACCCATTCACATCAATCTTG
cat	AGGGCAACTGGGATCTTACA	GATCCTTCAGGTGAGTCTGC
pink1	GGCAATGAAGATGATGTGGAAC	ATCACGTTGGGATGAGCACT
parkin	ATACCAGCACAGCATCAGCAG	GCACAGGAACTTGGCATTTG
ulk1b	AGGCCGAAAGTCTCACTTCA	AGCCATGTACATCGGAGACC
atg7	AGAGTCCAGTCCGATGTC	GAAGTAACAGCCGAGACG
atg12	TTTAAAGGCGGTCGGTGTCA	CCAACTTCTTGGTCCGGTGA
ambra1a	TAACCAGGAAACTGGCCAAC	AATATGCTGCAGGGGACAAC

beclin1	GTTCAGGTGGTCTGCGTTTT	GCAAACAGAAGCCAGTGTCA
lc3b	CCTCCAACTCAACTCCAACC	GCCGTCTTCGTCTCTTTCC
foxo3a	TTATGCCGCAAAGAAGAAAGC	CCGAACGAAAATCTGTCCAAG
mtor	AACCTACTGCCTCGACTTGC	CTCACAGCCACCACCAGTAG
ppargc1α	TATCAGTCCCAGTCGCATTCTC	TCATTCCCGTTTCTCATAGTCG
tsc1	GAAAAGAGAGGAAGGGGCG	GGATGAGTAAGGGCGGAAAG
prkaα1	ATGGAACCGCTACACCTCACC	GCAGACGCTTCGCCTTTTG
sesn2	TCCTTCCCGATTGTGTGTCG	GCCGTGATCCTCCCATGAAT
